# Adolescent mental health in Hong Kong: a longitudinal analysis of positive self‐evaluation, contextual influences and adverse experiences (2009–2014)

**DOI:** 10.1111/camh.70073

**Published:** 2026-02-18

**Authors:** Yanyan Ni, Ying Chen, Xiao Xiao, Corine S.M. Wong, Sunita M. Stewart, Michael Y. Ni

**Affiliations:** ^1^ School of Public Health, LKS Faculty of Medicine, The University of Hong Kong Hong Kong Special Administrative Region China; ^2^ Department of Epidemiology Harvard T.H. Chan School of Public Health Boston MA USA; ^3^ Department of Psychiatry UT Southwestern Medical Center Dallas TX USA; ^4^ State Key Laboratory of Brain and Cognitive Sciences, The University of Hong Kong Hong Kong Special Administrative Region China; ^5^ Urban Systems Institute, The University of Hong Kong Hong Kong Special Administrative Region China

**Keywords:** Positive self‐evaluation, positive environments, adverse experiences, mental well‐being, cohort studies

## Abstract

**Background:**

To examine whether positive self‐evaluation and contextual variables—such as those created by family, peers and schools—serve as promotive factors or protective factors in the context of adversity.

**Methods:**

Data were drawn from two waves of the youth survey of a population‐representative cohort in Hong Kong. Adolescents aged 10–14 (*n* = 3475) completed questionnaires about positive factors, adverse experiences and mental health at Wave 1 from March 2009 to April 2011, and 2696 of them were followed up at Wave 2 from August 2011 to March 2014. Those who completed both waves were included in the analysis (*n* = 2171).

**Results:**

Positive self‐evaluation, high family cohesion, positive peer relationship and positive school experience were statistically significantly associated with better behavioural well‐being at Wave 2, with incidence rate ratios (IRRs) of 1.01. Only positive self‐evaluation (IRR: 1.02, 95% CI 1.01–1.03) and positive school experience (IRR: 1.01, 1.00–1.02) were associated with better emotional well‐being in the fully adjusted models. We identified a statistically significant interaction between life stress and positive peer relationships in relation to behavioural well‐being (*p* = .009). The association between positive peer relationships and behavioural well‐being was only found among adolescents with ≥3 stressful events (IRR: 1.02, 1.01–1.04).

**Conclusion:**

Adolescents who possessed a positive sense of self and were nurtured in positive family, peer and school environments tended to exhibit better behavioural or emotional well‐being. Interventions should collaborate with families and schools to actively promote positive self‐evaluation and environments, with a particular emphasis on fostering positive peer relationships to enhance the health and well‐being of adolescents.

**Key Practitioner Message**

**What is currently known?**

- Positive childhood factors can serve as promotive factors that promote healthy development regardless of exposure to adversities. They can also function as protective factors that confer resilience in the context of adversity.
- There is a paucity of longitudinal studies that examine whether positive factors, such as positive self‐evaluation and environments created by family, peers and schools, and neighbourhoods, buffer against the negative impacts of adverse experiences on adolescent mental health.

**What has been shown?**

- Using two waves of longitudinal data and an ecological systems framework to select positive factors, we showed that positive self‐evaluation, high family cohesion, positive peer relationships and positive school experiences were associated with improved behavioural well‐being at Wave 2, while only positive self‐evaluation and positive school experiences were associated with emotional well‐being.
- While most positive factors directly promote positive outcomes, positive peer relationships may serve as a protective factor against the adverse effects of life stress on behavioural well‐being.

**What is the significance of this for clinical practice?**

- Our findings underscore the significance of positive self‐evaluation and environments as promotive factors and the protective role of positive peer relationships, thereby reinforcing the need for a comprehensive approach to childcare.
- Interventions should collaborate with families and schools to actively foster positive self‐evaluation and environments, with a particular emphasis on fostering positive peer relationships, to enhance health and well‐being of adolescents.

## Introduction

Adolescence represents a unique developmental period (Dick & Ferguson, 2015). Early adolescence, defined as ages 10–14 years, is one of the most neglected stages of development, despite its crucial role in shaping outcomes and behaviours in later adolescence and early adulthood (Blum, Astone, Decker, & Mouli, 2014). Many mental disorders, such as mood or anxiety disorders, typically originate in childhood or adolescence and peak at approximately 15 years (McGrath et al., 2023).

Early life adversities (e.g., family disruptions, physical fighting, bullying victimisation) are significant risk factors for long‐term health and behavioural problems among adolescents (LeMoult et al., 2020; Loeber, Green, Lahey, & Kalb, 2000; Middlebrooks, 2007; Moore et al., 2017; Sosin, Koepsell, Rivara, & Mercy, 1995). Notably, adverse childhood experiences (ACEs) only represent one end of the continuum of experiences occurring that influence the trajectory of health and development (Sege & Harper Browne, 2017).

From the perspective of developmental psychopathology, these positive experiences and contexts can serve as promotive factors that promote healthy development regardless of exposure to adversities (Masten, Lucke, Nelson, & Stallworthy, 2021; Narayan, Lieberman, & Masten, 2021). They can also function as protective factors, for instance, modifiers of the associations between ACEs and adolescent health or behaviours, as posited by the resilience theory that favourable life experiences can confer resilience in the context of adversity or negative experiences (Masten et al., 2021; Narayan et al., 2021). Different frameworks for preventing or mitigating the effects of ACEs have been proposed, including more recently—‘HOPE: Health Outcomes from Positive Experiences’—which emphasises the need to actively promote positive experiences and environments and proposes a holistic approach to optimal child health (Sege & Harper Browne, 2017). As such, a sole focus on either positive aspects or adverse experiences is not sufficient to achieve improved learning, behaviour, and physical and mental health outcomes in children and adolescents (Anderson et al., 2022; Bethell, Jones, Gombojav, Linkenbach, & Sege, 2019; Sege & Harper Browne, 2017).

An increasing number of studies have simultaneously evaluated both adverse and positive experiences recently (Narayan et al., 2021). However, most studies are cross‐sectional in design (Elmore, Crouch, & Kabir Chowdhury, 2020; Qu et al., 2022), which do not allow for directional inferences or rely on retrospective reports of adult populations, potentially introducing recall bias (Bethell et al., 2019; Han, Dieujuste, Doom, & Narayan, 2023; Hou et al., 2022). There is a paucity of longitudinal studies that examine the coexistence and interplay of positive factors and adverse experiences and how they may be related to later mental health in adolescents (Wang, Jiang, Yang, & Choi, 2021). Such studies are important for understanding the nuanced relationships between these experiences and their long‐term effects.

We aimed to utilise two waves of longitudinal data collected from adolescents aged 10–14 to examine the individual associations of positive self‐evaluation and contextual variables—such as those created by family, peers and schools—and adverse experiences with mental health in adolescents and to assess whether these positive factors serve as protective factors against the harmful impacts of adversity (Figure 1). The selection of positive factors was drawn from previous research and the Resiliency Theory grounded in Bronfenbrenner's ecological systems framework, suggesting that multiple systems (e.g., individual, family, peer, schools, neighbourhoods) interact to affect the course of development and that resilience itself is constantly evolving within individuals and systems (Bellis et al., 2018; Bronfenbrenner, 1977; Narayan et al., 2021; Narayan, Rivera, Bernstein, Harris, & Lieberman, 2018; Sege & Harper Browne, 2017; Slopen, Chen, Guida, Albert, & Williams, 2017). Our hypothesis was that a positive sense of self and contextual variables at Wave 1 would be associated with better mental health at Wave 2. Conversely, we also hypothesised that adverse experiences at Wave 1 would be associated with worse mental health at Wave 2 and these associations would be mitigated by the presence of positive factors.

**Figure 1:**
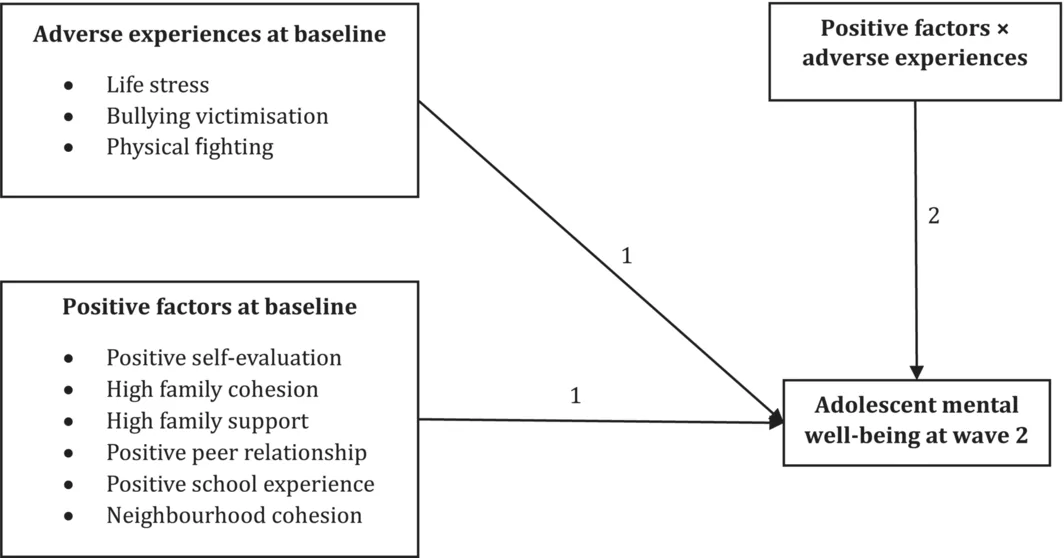
Conceptual models showing the relationship between positive factors, adverse experiences and adolescent mental health. Pathway 1 tested the individual associations of baseline positive factors and adverse experiences with adolescent mental health at Wave 2; pathway 2 tested the moderation effect of positive factors through interaction terms with adverse experiences. Full models adjusted for baseline covariates, including child age and sex, parental education, employment, marital status, housing type and household income, and baseline mental well‐being

## Methods

### Study design and participants

Our participants were drawn from the FAMILY Cohort, a prospective population‐based cohort study in Hong Kong. The sample was obtained with stratified random sampling of households from all 18 districts with sample sizes proportionate to the population of each district (Leung et al., 2017). Data collection for Wave 1 was between March 2009 and April 2011. From all households enrolled in the cohort, adolescents aged 10–14 (*n* = 3475) were invited to complete a questionnaire about their life experiences and health. Wave 2 took place between August 2011 and March 2014, with all participants invited to take part, among which 2696 (78%) adolescents (age range 11–19 years) were successfully followed up (Figure 2). Participants who completed the questionnaire at both waves were included for the final analysis (*n* = 2171; age range at Wave 2: 11–17 years). The STROBE guidelines were used to ensure the transparent reporting of this cohort study (Table S1).

**Figure 2:**
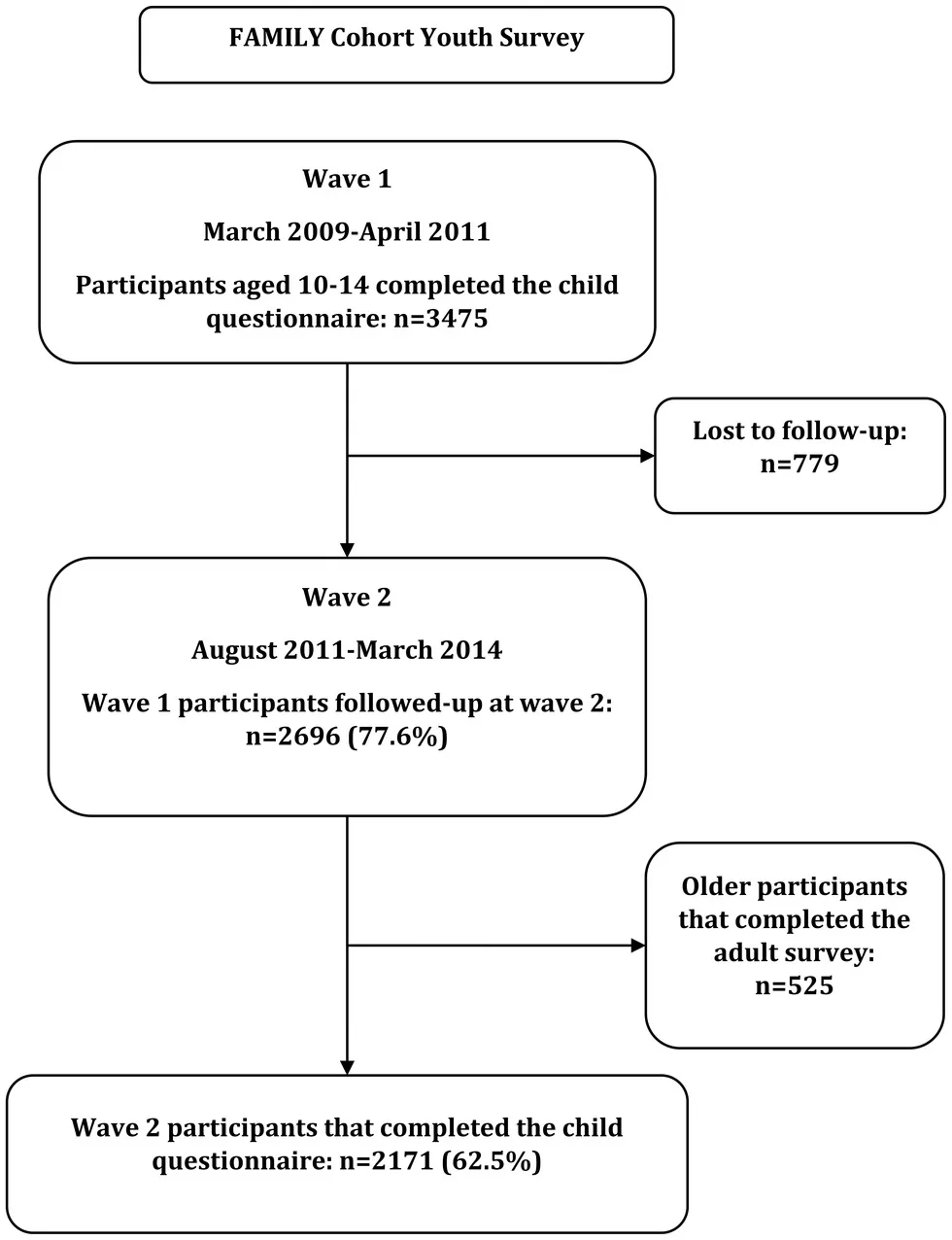
Flowchart of adolescent participants in the FAMILY Cohort

Our analyses were exploratory in nature and not preregistered, but they were guided by the established ecological systems framework, resilience theory and prior research, which provided a structured foundation for our investigations. This ensured that our exploratory approach was informed and methodologically sound, even in the absence of preregistration.

### Ethics

Ethical approval was obtained from the Institutional Review Board of The University of Hong Kong/Hospital Authority Hong Kong West Cluster (Wave 1 reference number: UW 09‐387; Wave 2 reference number: UW 11‐332). Written parental consent was obtained for the surveyed adolescents. Approval covered data collection and data analysis for this study.

### Outcomes

*Mental health at Wave 2* was measured from two aspects: behavioural and emotional well‐being, using the behaviour (16 items) and mental health (16 items) scales of the Child Health Questionnaire Child Form 87 Questions (CHQ‐CF87), respectively (Landgraf et al., 1998). The CHQ‐CF87 was designed for children aged 10 or older. The Chinese version is robust and reliable for use in Chinese populations (Ng, Landgraf, Chiu, Cheng, & Cheung, 2005). Cronbach alpha coefficients were .90 for the behaviour scale and .91 for the mental health scale in Ng et al. (2005)'s study. The behaviour scale is designed to measure overt behaviour as a dimension of mental health, covering aggression, delinquency, hyperactivity/impulsivity and social withdrawal. Participants were asked to respond to statements that describe behaviours (e.g., difficulty in paying attention, arguing, cheating and lying) and ability to get along with others in the past 4 weeks using a 5‐point scale from 1 = *very often to* 5 = *never*. The mental health scale measures feelings of anxiety and depression (e.g., feeling sad, lonely, unhappy, worried and nervous) that are an affective component of mental health (i.e., emotional well‐being). Frequency is captured using a 5‐level continuum ranging from 1 = *all of the time* to 5 = *none of the time* in the past 4 weeks. Scores were summed up and transformed to a 0–100 scale, with a higher score indicating better behavioural and emotional well‐being. In our sample, Cronbach alpha coefficients were .85 for the behaviour scale and .87 for the mental health scale.

### Baseline exposures and covariates

*Positive factors* included six components: positive self‐evaluation, high family cohesion, high family support, positive peer relationship, positive school experience and neighbourhood cohesion. The selection of components was guided by Bronfenbrenner's framework, and six variables were created (Table S2). Self‐evaluation, peer relationship and school experiences were assessed via the following questions: ‘How good or bad have you felt about yourself in the last four weeks,’ ‘How was your friendship in the past four weeks’, and ‘How was your school work in the past four weeks’. Response scales ranged from 1 = *very bad* to 5 = *very good*. A higher score indicates the tendency towards positive self‐evaluation, positive peer relationship and positive school experience.

The other three positive factors were measured using validated scales: self‐reported family cohesion by the 9‐item cohesion subscale of the Family Environment Scale (FES) with responses ranging from 1 = *strongly agree* to 6 = *strongly disagree* (Lee, Wong, Chow, & McBride‐Chang, 2006; Moos, 1990), self‐reported family support by the 5‐item Family APGAR (i.e., adaptation, partnership, growth, affection and resolve) with responses ranging from 0 = *hardly ever* to 2 = *almost always* (Nan, Lee, Ni, Chan, & Lam, 2013; Smilkstein, Ashworth, & Montano, 1982) and parent‐reported neighbourhood cohesion by the 4‐item Neighbourhood Social Cohesion Scale with responses ranging from 1 = *strongly agree* to 5 = *strongly disagree* (Sampson, Raudenbush, & Earls, 1997). For each of these three scales, all items were summed and averaged to create a continuous score with a higher score indicating greater support or cohesion. Scores range 1–6 for family cohesion, 0–2 for family support and 1–5 for neighbourhood cohesion.

The FES cohesion subscale assesses the intensity with which family members are perceived as concerned, committed and supportive of the adolescent (Moos, 1990). A Chinese version has been used in adolescents aged 14–17 in Lee et al. (2006)'s study and the Cronbach alpha coefficient was .86 in their sample. In their study, cohesion was correlated with suicide ideation (*r* = −.44), depression (*r* = −.46) and family support (*r* = .75), which provided evidence for construct validity. In our sample, the Cronbach alpha coefficient was .87. The Family APGAR collects information on satisfaction with the aforementioned five areas of family function. Evidence from various population groups, including Chinese populations, has supported its validity and reliability (Smilkstein et al., 1982). In our sample, the Cronbach alpha coefficient was .92.

For neighbourhood cohesion, the four items have been shown reliable in previous studies (Mujahid, Roux, Morenoff, & Raghunathan, 2007). Example items include ‘people around here are willing to help their neighbours’ and ‘this is a close‐knit neighbourhood’. The original validation was based on a survey in the US (Sampson et al., 1997). We omitted the fifth item (people in this neighbourhood do not share the same values) from the original list given its poor fit (item‐test and item‐rest correlations of .47 and .22). Item‐test correlations are the correlations between each item and the total scale score, reflecting how well the item aligns with the overall construct. Item‐rest correlations are similar but exclude the item itself, estimating its consistency with the other items. These metrics help assess each item's contribution to the scale's reliability and validity. While the neighbourhood cohesion scale has not been formally validated in Hong Kong, it has been previously used in a local setting yielding a Cronbach alpha coefficient of .77 (Chou, 2012). In the current sample, the four items exhibited a Cronbach alpha coefficient of .87.

*Adverse experiences* assessed in this study included life stress, school bullying victimisation and physical fighting. Life stress was measured using the Life Event Checklist (Wong, Stewart, Ho, Rao, & Lam, 2005), which is the most commonly used approach to measuring stressful events (Cohen, Murphy, & Prather, 2019). Participants were asked whether they had experienced or were bothered by a list of 14 stressful events in the past 12 months (Table S3), including death of close relative or friend, hospitalisation due to illness or accident, victim of violence, increased arguments with parents and worsened family financial situation. These items were selected for their relevance in the context of Hong Kong. Validity is supported by correlations of .35, .46 and .37 for this scale with anxiety symptoms, depressive symptoms and unpleasant family relationships, respectively, in the larger Hong Kong sample (Wong, Ho, & Lam, 2004). Cronbach alpha coefficient was .88 in the present sample. Life stress was coded using cumulative score groupings of 0, 1, 2 and ≥ 3 events.

Given the increasing levels of bullying and aggression among adolescents in Hong Kong (OECD, 2019), we also examined school bullying victimisation and physical fighting as two other indicators of adverse experiences. School bullying victimisation was measured using a question adopted from the 2009 Youth Risk Behaviour Survey (YRBS) (Eaton et al., 2010; Lee, Tsang, Lee, & To, 2001). Participants were asked whether they had been bullied on school property during the past 12 months. Answers of ‘yes’ indicated experience of school bullying victimisation. Physical fighting was measured using two questions from the 2009 YRBS (Eaton et al., 2010; Lee et al., 2001). Participants were asked (1) whether they had been in a physical fight or (2) whether they were injured in a physical fight and had to be treated by a doctor or nurse during the past 12 months. Participants with answers of ‘yes’ to any of the two questions were considered to have been in a physical fight.

*Covariates at baseline* included child age and sex, parental education (primary, secondary and tertiary), parent employment (employed, potentially employed and unemployed), parent marital status (married and others), household income (<10,000, 10,000–19,999, 20,000–39,999 and ≥40,000 HKD), housing type (public rental housing, subsidised home ownership scheme housing and private permanent housing) and baseline behavioural and emotional well‐being measured using the CHQ‐CF87. The choice of covariates is guided by previous methodological and empirical research (Bellis et al., 2018; Bethell et al., 2019; Qu et al., 2022; VanderWeele, Jackson, & Li, 2016). A directed acyclic graph (DAG) illustrating this and our analytic strategy is provided in Figure S1.

### Statistical analysis

We analysed our outcomes of behavioural well‐being and emotional well‐being separately. We also conducted separate analyses for the six components of positive factors (continuous scores) and the three components of adverse experiences (categorical variables; Figure 1). For each component of baseline positive factors, we used Poisson regression to examine its longitudinal associations with behavioural well‐being at Wave 2 given the skewed distribution in the outcome (model fit statistics reported in Table S4). For emotional well‐being, we initially employed Poisson regression due to the skewed distribution of the outcome variable. However, the goodness‐of‐fit chi‐square tests indicated that the use of Poisson regression was inappropriate (*p* values < .001). Upon identifying significant overdispersion in the outcome, we transitioned to using negative binomial regression (model fit statistics reported in Table S5). We first reported incidence rate ratios (IRRs) and their 95% confidence intervals (CIs) from unadjusted models (Model 1), then controlled for baseline sociodemographic variables (Model 2) and further adjusted for baseline mental health (Model 3). All models adjusted for clustering effects within the same household through a clustered sandwich estimator for the standard errors specified by the vce option in Stata. Similar analyses were performed for each component of adverse experiences. To test the protective processes of positive factors (i.e., whether they modified the associations between exposure to stressful events and outcomes), we added their interaction terms with adverse experiences to the established models (Figure 1). Following the association results, life stress was further categorised into binary variables for the interaction analysis: (1) for behavioural well‐being: <3 versus ≥3 events; (2) for emotional well‐being: <2 versus ≥2 events. Following the detection of statistically significant interaction effects, subgroup analyses were performed using dichotomised positive factors to facilitate interpretability. To account for demographic differences between the survey sample and the underlying population, we applied raking and inverse probability of censoring weighting to the data (Appendix S1). Inverse probability weighting was used to account for potential attrition bias in a prospective cohort study. The censoring weights were defined as the inverse of the probability of participating in the study at Wave 2, estimated using logistic regression with baseline characteristics. Raking was then applied so that each wave would be representative of the studied population. We applied multiple imputation to handle incomplete data on covariates and pooled the results from 20 imputed datasets using Rubin's rules (Rubin, 2004). Results were similar before and after imputation (Tables S6 and S7), so imputed results are presented in the main text. All analyses were conducted in Stata/MP 17.0, and the corresponding analysis syntax has been provided in Appendix S2.

## Results

### Descriptive characteristics

In our analytic sample, 52.4% were boys, 16.4% had parents of primary education, and 50.3% lived in public rental properties. The mean follow‐up was 825 days (range 478–1784 days). The drop‐out analysis indicated that assessed participants were slightly younger than nonassessed participants at Wave 2 (11.9 vs. 12.5 years), but differences in other sociodemographics and exposures were small (Cohen's *w* 0.00–0.08; Table 1). In the past 12 months, over three‐quarters (28.8%) of participants reported having three or more stressful life events, 7.8% reported being bullied on school property, and 19.8% being involved or injured in a physical fight. Mean scores (*SD*) of positive factors were 3.6 (0.7) for self‐evaluation, 4.6 (0.8) for family cohesion, 1.3 (0.6) for family support, 4.1 (0.8) for school experience and 3.5 (0.8) for peer relationship. Median scores (interquartile range) of behavioural well‐being and emotional well‐being at Wave 1 were 90 (82–95) and 85 (76–93), respectively. Median scores (interquartile range) of behavioural well‐being and emotional well‐being at Wave 2 were 94 (87–97) and 87 (78–93), respectively.

**Table 1:** Drop‐out analysis of Wave 1 sample assessed at Wave 2

Wave 1 variables		Assessed at Wave 2	Not assessed at Wave 2	Cohen's effect size
		*n* = 2171	*n* = 1304	
Child age	Mean (*SD*)	11.9 (1.4)	12.5 (1.3)	0.47
Male sex	% (*n*/*N*)	52.4 (1138/2171)	52.4 (683/1304)	0.00
Parental education				
Primary	% (*n*/*N*)	16.4 (347/2116)	18.4 (231/1254)	0.04
Secondary	% (*n*/*N*)	69.2 (1465/2116)	69.2 (868/1254)	
Tertiary	% (*n*/*N*)	14.4 (304/2116)	12.4 (155/1254)	
Parental employment				
Unemployed	% (*n*/*N*)	2.8 (60/2117)	1.8 (23/1256)	0.04
Potential employed	% (*n*/*N*)	40.1 (849/2117)	38.2 (480/1256)	
Employed	% (*n*/*N*)	57.1 (1208/2117)	60.0 (753/1256)	
Parental marital status				
Married	% (*n*/*N*)	88.0 (1859/2113)	87.3 (1095/1254)	0.01
Other	% (*n*/*N*)	12.0 (254/2113)	12.7 (159/1254)	
Monthly household income (HKD)				
<10,000	% (*n*/*N*)	28.5 (595/2085)	29.6 (362/1222)	0.02
10,000**–**19,999	% (*n*/*N*)	35.8 (747/2085)	36.3 (444/1222)	
20,000**–**39,999	% (*n*/*N*)	23.6 (493/2085)	23.5 (287/1222)	
≥40,000	% (*n*/*N*)	12.0 (250/2085)	10.6 (129/1222)	
Housing type				
Public rental housing	% (*n*/*N*)	50.3 (1073/2135)	54.2 (685/1263)	0.05
Subsidised home ownership scheme housing	% (*n*/*N*)	19.1 (408/2135)	15.4 (194/1263)	
Private permanent housing	% (*n*/*N*)	30.6 (654/2135)	30.4 (384/1263)	
Scores of positive factors				
Positive self‐evaluation	Mean (*SD*)	3.6 (0.7)	3.6 (0.7)	0.00
High family cohesion	Mean (*SD*)	4.6 (0.8)	4.5 (0.7)	0.08
High family support	Mean (*SD*)	1.3 (0.6)	1.3 (0.6)	0.08
Positive peer relationship	Mean (*SD*)	4.1 (0.8)	4.0 (0.7)	0.02
Positive school experience	Mean (*SD*)	3.5 (0.8)	3.5 (0.8)	0.01
Neighbourhood cohesion	Mean (*SD*)	3.6 (0.5)	3.6 (0.5)	0.00
Stressful experiences				
1 event	% (*n*/*N*)	15.2 (331/2171)	17.0 (222/1304)	0.06
2 events	% (*n*/*N*)	15.1 (328/2171)	11.5 (150/1304)	
≥3 events	% (*n*/*N*)	28.8 (626/2171)	27.8 (362/1304)	
Prevalence of school bullying victimisation	% (*n*/*N*)	7.8 (170/2171)	5.2 (68/1304)	0.05
Prevalence of physical fighting	% (*n*/*N*)	19.6 (425/2171)	15.3 (199/1304)	0.05
Behavioural well‐being score	Median (IQR)	90 (82–95)	91 (84–96)	0.08
Emotional well‐being score	Median (IQR)	85 (76–93)	85 (75–93)	0.00

Cohen's effect size = small 0.1; medium 0.3; large 0.5. IQR, interquartile range.

### Promotive processes

In Model 1 (unadjusted models), positive self‐evaluation, high family cohesion, high family support, positive peer relationship and positive school experience were statistically significantly associated with better behavioural well‐being at Wave 2 (Table 2; IRRs range: 1.02–1.03). Associations remained statistically significant in Model 2 after adjusting for baseline sociodemographics. Positive self‐evaluation, high family cohesion, positive peer relationship and positive school experience remained statistically significant in Model 3 after further adjusting for baseline behavioural well‐being with IRRs of 1.01 (all *p* values < .05). Results were similar for emotional well‐being in unadjusted models, but only positive self‐evaluation (Table 3; IRR: 1.02, 95% CI 1.01–1.03) and positive school experience (IRR: 1.01, 95% CI 1.00–1.02, *p* = .034) remained statistically significant in the fully adjusted models. The association between positive peer relationship and emotional well‐being was not statistically significant, with a *p*‐value of .05. Associations were nonsignificant for neighbourhood cohesion in all models.

**Table 2:** Longitudinal associations between baseline exposures and behavioural well‐being at Wave 2 using Poisson regression

	Model 1	Model 2	Model 3
	IRR (95% CI)	IRR (95% CI)	IRR (95% CI)
Positive experience scores			
Positive self‐evaluation	**1.03 (1.02, 1.04)**	**1.03 (1.02, 1.03)**	**1.01 (1.00, 1.02)**
High family cohesion	**1.02 (1.01, 1.03)**	**1.02 (1.01, 1.03)**	**1.01 (1.00, 1.02)**
High family support	**1.02 (1.01, 1.03)**	**1.02 (1.01, 1.03)**	1.01 (1.00, 1.02)
Positive peer relationship	**1.02 (1.01, 1.02)**	**1.02 (1.01, 1.02)**	**1.01 (1.00, 1.02)**
Positive school experience	**1.03 (1.02, 1.03)**	**1.02 (1.02, 1.03)**	**1.01 (1.01, 1.02)**
Neighbourhood cohesion	1.00 (0.99, 1.01)	1.00 (0.99, 1.01)	1.00 (0.99, 1.01)
Stressful life events (ref. = no)			
1 event	1.00 (0.99, 1.02)	1.00 (0.99, 1.02)	1.00 (0.99, 1.02)
2 events	1.00 (0.98, 1.01)	1.00 (0.98, 1.01)	1.00 (0.99, 1.02)
≥3 events	**0.97 (0.96, 0.99)**	**0.97 (0.96, 0.99)**	1.00 (0.98, 1.01)
School bullying victimisation (ref. = no)	**0.96 (0.94, 0.98)**	**0.96 (0.94, 0.98)**	0.99 (0.96, 1.01)
Physical fighting (ref. = no)	**0.98 (0.97, 0.99)**	**0.98 (0.97, 0.99)**	0.99 (0.98, 1.01)

Positive factors were analysed as continuous scores. Model 1: unadjusted. Model 2: adjusted for child age and sex, parental education, employment, marital status, housing type and household income at Wave 1. Model 3: further adjusted for behavioural well‐being at Wave 1. Bold fonts indicate *p* values <.05. Results with multiple imputations for missing data are presented (*N* = 2171). IRR, incidence rate ratio.

**Table 3:** Longitudinal associations between baseline exposures and emotional well‐being at Wave 2 using negative binomial regression

	Model 1	Model 2	Model 3
	IRR (95% CI)	IRR (95% CI)	IRR (95% CI)
Positive factor scores			
Positive self‐evaluation	**1.04 (1.03, 1.05)**	**1.04 (1.03, 1.05)**	**1.02 (1.01, 1.03)**
High family cohesion	**1.03 (1.02, 1.04)**	**1.03 (1.02, 1.04)**	1.01 (1.00, 1.02)
High family support	**1.03 (1.01, 1.05)**	**1.03 (1.01, 1.04)**	1.00 (0.98, 1.01)
Positive peer relationship	**1.02 (1.01, 1.04)**	**1.02 (1.01, 1.04)**	1.01 (1.00, 1.02)
Positive school experience	**1.03 (1.02, 1.04)**	**1.02 (1.01, 1.04)**	**1.01 (1.00, 1.02)**
Neighbourhood cohesion	1.01 (0.99, 1.02)	1.01 (0.99, 1.02)	1.00 (0.99, 1.01)
Stressful life events (ref. = no)			
1 event	0.99 (0.97, 1.02)	1.00 (0.97, 1.02)	1.00 (0.98, 1.02)
2 events	**0.97 (0.95, 1.00)**	**0.98 (0.96, 1.00)**	0.99 (0.97, 1.01)
≥3 events	**0.96 (0.94, 0.98)**	**0.97 (0.95, 0.99)**	1.01 (0.99, 1.03)
School bullying victimisation (ref. = no)	0.97 (0.94, 1.01)	**0.96 (0.93, 1.00)**	1.00 (0.96, 1.03)
Physical fighting (ref. = no)	0.99 (0.97, 1.01)	**0.98 (0.96, 1.00)**	0.99 (0.97, 1.01)

Positive factors were analysed as continuous scores. Model 1: unadjusted. Model 2: adjusted for child age and sex, parental education, employment, marital status, housing type and household income at Wave 1. Model 3: further adjusted for emotional well‐being at Wave 1. Bold fonts indicate *p* values <.05. Results with multiple imputations for missing data are presented (*N* = 2171). IRR, incidence rate ratio.

### Protective processes

In models 1 and 2, exposure to ≥3 stressful events, bullying victimisation at school, and physical fighting were all associated with poorer behavioural well‐being (Table 2; IRRs range: 0.95–0.98). Exposure to 2 or ≥3 stressful events was associated with poorer emotional well‐being (Table 3; IRRs range: 0.96–0.98). All associations became nonsignificant in Model 3 after further adjusting for baseline mental health. Analyses testing protective processes of positive factors identified a statistically significant interaction between life stress (≥3 stressful events vs. <2 stressful events) and positive peer relationship scores in relation to behavioural well‐being (*p* = .009). Our subgroup analysis showed that the association between life stress and behavioural well‐being was nonsignificant in both adolescents with (IRR: 1.00, 95% CI 0.99–1.01) and without (IRR: 0.98, 95% CI 0.95–1.01) positive peer relationship (scores of 4–5 vs. scores of 1–3). Adolescents facing three or more stressful events who maintained positive peer relationships exhibited, on average, the highest behavioural well‐being scores (Figure 3; predictive marginal mean: 90.6, 95% CI 89.7–91.5), whereas those with similar stress exposure but no positive peer relationships displayed the lowest scores (87.7, 95% CI 85.4–90.0). Further analysis showed a statistically significant association between positive peer relationship and behavioural well‐being among adolescents with exposure to ≥3 stressful events (IRR: 1.02, 95% CI 1.01–1.04) but a null association among adolescents with exposure to <2 stressful events (IRR: 1.00, 95% CI 0.99–1.01). We did not find any other statistically significant interactive effects between positive factors and adverse experiences (i.e., life stress, bullying, and physical fighting) in relation to behavioural and emotional well‐being (Table S8).

**Figure 3:**
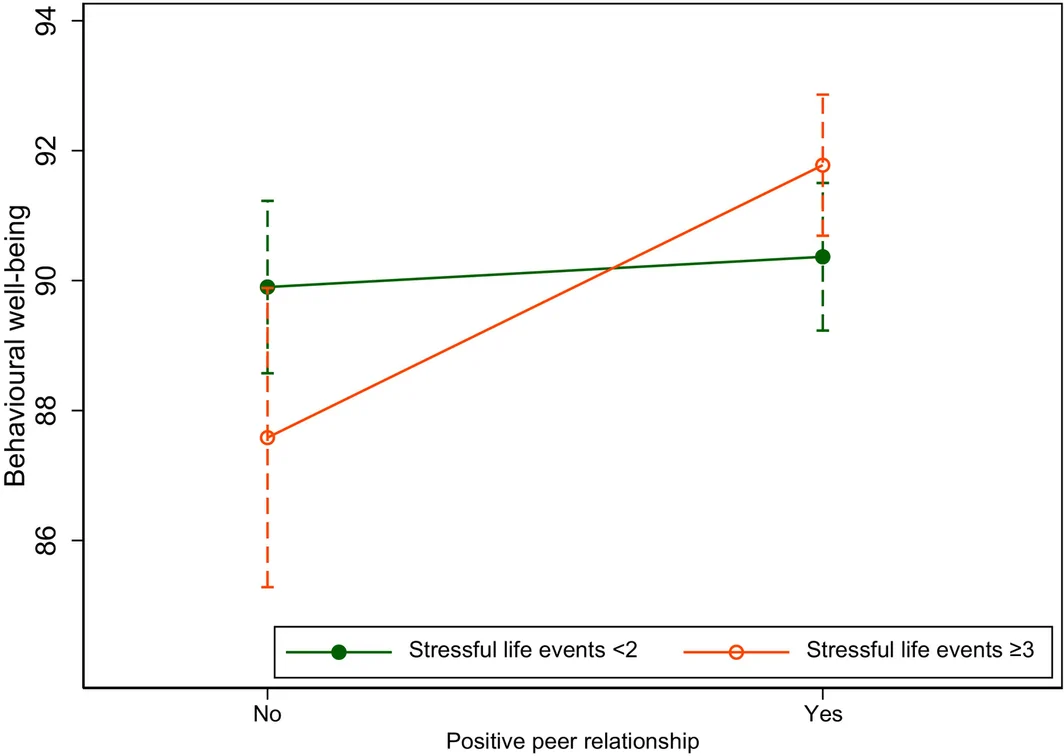
Predictive margins and 95% confidence intervals for each of the levels of interaction between life stress and positive peer relationship in relation to behavioural well‐being. Model adjusted for child age and sex, parental education, employment, marital status, housing type and household income at Wave 1, and behavioural well‐being at Wave 1. Imputed results are presented. Subgroup analyses using categorical cut‐offs were performed to facilitate interpretability following the detection of statistically significant interaction effects. Peer relationship scores were converted into a binary variable to indicate the presence of positive peer relationship as follows: (1) ‘yes’ for scores of 4 (*somewhat good*) and 5 (*very good*); (2) ‘no’ for scores of 1 (*very bad*), 2 (*somewhat bad*) and 3 (*neither good nor bad*)

## Discussion

Utilising two waves of longitudinal data and an ecological systems framework to select positive factors, we showed evidence that positive sense of self and positive contextual variables at different levels, such as cohesive family members, favourable peer relationships or school experiences, were prospectively associated with better behavioural well‐being in adolescents, independent of baseline behavioural well‐being, while for emotional well‐being, only positive sense of self and positive school experiences remained statistically significant in the fully adjusted models that additionally adjusted for baseline emotional well‐being. The effect sizes in our study appeared small, likely influenced by the use of continuous outcomes as no established cut‐offs exist for CHQ‐CF87 and the fact that most adolescents reported relatively high well‐being scores on average. Nonetheless, these effects can still carry significant and meaningful implications for promoting optimal mental well‐being when applied across an entire population (Carey, Ridler, Ford, & Stringaris, 2023; VanderWeele, Mathur, & Chen, 2019). For example, a one‐point change in the total score of a mental health measure might appear negligible to clinicians or trialists when assessing individual‐level outcomes. However, at the population level, such a seemingly small shift can lead to a substantial change in the absolute number of individuals crossing the threshold for mental health conditions (Carey et al., 2023). Conversely, adverse experiences, such as life stress (e.g., family dysfunction, increased conflicts with parents), bullying victimisation and physical fighting, were associated with worse adolescent mental health, but these associations were no longer statistically significant after further adjustments for baseline mental health.

Notably, our analyses indicated different patterns for association results which vary with types of positive factors and subscales of mental health outcomes examined. A direct comparison with the existing literature is challenging as prior research predominantly focuses on the emotional dimension of mental health and uses a composite measure to assess positive experiences (Han et al., 2023). To illustrate, positive self‐evaluation and positive school experiences, but not other positive factors, were associated with both behavioural and emotional well‐being, which correspond largely to externalising and internalising behaviours. Externalising behaviours refer to outwardly directed behaviour problems, such as rule‐breaking actions, aggression and delinquency, whereas internalising behaviours are characterised by anxiety and depression (Achenbach & Edelbrock, 1978). Positive perceptions of oneself and academic abilities can foster healthy self‐esteem and self‐worth (Brown, Dutton, & Cook, 2001), which have been associated with better mental health, fewer depressive and anxiety symptoms, and a lower risk of externalising problems (Arsandaux, Galera, & Salamon, 2021; Birkeland, Melkevik, Holsen, & Wold, 2012; Donnellan, Trzesniewski, Robins, Moffitt, & Caspi, 2005; Scott & Dearing, 2012). Further, we observed that the components of high family cohesion and positive peer relationships were statistically significant only for behavioural well‐being but not emotional well‐being. It may be that cohesive families can provide positive role models, shape self‐regulation and teach conflict resolution, which can deter adolescents from engaging in risky or disruptive behaviours (Dishion, 1990; Eisenberg, Spinrad, & Eggum, 2010; Fosco, Caruthers, & Dishion, 2012). The association between positive peer relationships and emotional well‐being did not reach statistical significance (*p* = .05), which may be due to the small sample size.

Our findings can be interpreted through the lens of developmental psychopathology, providing support for the importance of promotive processes associated with positive childhood experiences. Therefore, to enhance adolescent mental health, it is crucial to promote positive self‐evaluation, school experiences, and family cohesion. These components should be incorporated into interventions aimed at promoting optimal health among adolescents. While most positive factors directly promote positive outcomes, positive peer relationships were found to act as a protective factor against the negative impacts of life stress on behavioural well‐being, which partially aligns with our hypothesis and the resilience theory. Nevertheless, we did not identify other statistically significant interactive effects, underscoring the importance of conducting more longitudinal studies to explore alternative sources of resilience following adversity (Fritz, de Graaff, Caisley, van Harmelen, & Wilkinson, 2018; Han et al., 2023; VanBronkhorst et al., 2024).

Interestingly, adolescents with three or more stressful events and positive peer relationships showed optimal behavioural well‐being, indicating that those experiencing more life stress were also more susceptible or plastic, in that they were more influenced than those with less stress by positive peer relationships. This provides support for the differential susceptibility theory which proposes that the same individuals who are more vulnerable to negative environments are also more likely to benefit from positive environments (Ellis, Boyce, Belsky, Bakermans‐Kranenburg, & van Ijzendoorn, 2011). Our finding indicates that interventions and treatments of mental health problems may benefit from incorporating differential susceptibility thinking (Assary, Krebs, & Eley, 2023).

We controlled for baseline covariates and outcomes to mitigate the risk of reverse causality in our analysis (VanderWeele et al., 2016). Notably, several associations became nonsignificant in the fully adjusted models. In our study, baseline mental health was assessed based on experiences in the past 4 weeks. Self‐evaluation, peer relationships and school experiences were also measured within a timeframe of 4 weeks, while stressful life events, bullying victimisation and physical fighting were measured based on experiences within the past 12 months. Considering the temporal sequence, it is possible that baseline mental health may act as a mediator rather than a confounding factor (Figure S2). It can also be that baseline mental health serves as a moderator in the associations between positive factors and mental health at Wave 2. For instance, adolescents with poorer baseline mental health benefit differently from positive factors compared with those with better baseline mental health. We were able to test the moderator and mediator models (Appendix S1). Our exploratory analysis did not provide support for baseline mental health as the moderator. However, it revealed a strong mediating effect of baseline mental health on the association between baseline positive factors and mental health outcomes at Wave 2 (Tables S9 and S10). Replication in other samples is required to confirm the mediating or moderating role of baseline mental health and draw conclusions from the results.

Consistent with prior work, we showed that exposure to stressful events such as being bullied on school property and physical fighting were associated with poorer mental health, despite the associations mediated by baseline mental health (Table S9). The Life Event Checklist in our study assessed relatively short‐lived stress that may be tolerable (e.g., hospitalisation due to illness or accident), more severe and prolonged stress (e.g., the death of a loved one, parental separation or divorce), and intense stress that can be toxic if frequent (e.g., repeated exposure to violence) (Middlebrooks, 2007). We did not distinguish among different types of stress in the analysis given the difficulty in judging the frequency, intensity and duration of each stressful event. Both school bullying and physical fighting are important international concerns. In our survey conducted between 2009 and 2011, the prevalence of bullying victimisation was 7.8%. According to the 2018 survey data from the Programme for International Students Assessment, nearly 30% of Hong Kong students reported being bullied at least a few times a month, much higher than other jurisdictions. For instance, the corresponding numbers were 13% in Taiwan, 18% in mainland China, 26% in the US and 23% across OECD countries (OECD, 2019). In our survey, 20% of Hong Kong adolescents reported being involved or injured in a physical fight, while in the US, 33% had been in a physical fight in 2011 and 18% in 2021 (US Centers for Disease Control and Prevention, n.d.). Drawing from this study and the growing evidence, prevention of youth violence (i.e., bullying, physical fighting) should be a priority for public health and educational systems in Hong Kong and internationally. Victims of bullying and adolescents involved in physical violence should also be prioritised for ongoing and targeted interventions by clinicians and violence prevention specialists.

Neighbourhood cohesion, reported by parents, was unrelated to any of the outcomes, in contrast to previous research (Kingsbury et al., 2015) and the ecological framework supporting that community‐level factors play a role in child and adolescent development (Bronfenbrenner, 1977; Cicchetti & Lynch, 1993). One potential explanation is that although parents may report high social cohesion, their children may have had different experiences. Children may spend more time in school, and thus, the influence of the neighbourhood may be diminished.

Our study has several limitations. First, positive experiences in our study were measured and analysed as separate domains. Researchers have recently developed a Benevolent Childhood Experiences scale as a retrospective and composite measure of positive childhood experiences at multiple levels, encompassing internal and familial factors, relationships with friends, at school and in the community (Narayan et al., 2018). Nevertheless, our analyses indicated that associations with health and well‐being varied with types of positive experiences. While valuable to test cumulative benefits, using a composite score may overlook specific associations of each type of positive experiences with mental health outcomes in adolescents. Second, cohort studies are susceptible to selection and attrition bias. Our drop‐out analysis indicated that assessed and nonassessed adolescents at Wave 2 had minimal differences in sociodemographics and exposures. Further, the application of censoring weights, raking and multiple imputation enhanced our research findings (Fitzmaurice, Laird, & Ware, 2012; Howe, Cole, Lau, Napravnik, & Eron Jr., 2016). Third, data on exposures and outcomes were mostly self‐reported by adolescents. Future research should consider collecting information from multiple informants (e.g., parents, teachers). Fourth, the mental health component of CHQ‐CF87 is a combined measure of anxiety and depressive feelings. As such, we were not able to distinguish whether the associations would be similar or different for anxiety and depression separately. In addition, outcomes were analysed as continuous scores as no established cut‐offs are available for CHQ‐CF87, which may have contributed to the reported relatively small effect sizes. Lastly, given the nature of the observational data, our findings should be interpreted as associations rather than causal relationships. In line with the methodological guidance of VanderWeele et al. (2016), we adjusted for baseline covariates and baseline mental well‐being to provide some evidence for causal inference. However, the availability of only two waves of data limited our ability to control for prior exposure or time‐varying exposures, which restricts the strength of causal inference. Further experimental studies and well‐designed longitudinal studies with control for prior exposure and outcome would be valuable to confirm these causal pathways (VanderWeele et al., 2016).

In conclusion, our study underscores the significance of positive self‐evaluation and nurturing family and school environments as promotive factors for adolescent behavioural and emotional well‐being, while positive peer relationships function as protective factors against the negative impacts of adversities. It is concerning that these positive factors have not been integrated into policy initiatives or practical applications, thereby reinforcing the need for a comprehensive approach to childcare, as outlined by the HOPE framework. Policies and interventions should collaborate with families and schools to actively foster positive self‐evaluation and positive environments at multiple levels, with a particular emphasis on cultivating positive peer relationships, to enhance health and well‐being of children and adolescents. Further longitudinal studies are required to identify alternative sources of resilience to prevent or mitigate the negative consequences following childhood adversity (VanBronkhorst et al., 2024).

## Conflict of interest statement

The authors have declared that they have no competing or potential conflicts of interest.

## Funding information

The youth mental health studies of the FAMILY Cohort were funded by the Hong Kong Jockey Club Charities Trust from 2007 to 2014 and from 2024 to 2028.

## Ethical considerations

Ethical approval was obtained from the Institutional Review Board of the University of Hong Kong/Hospital Authority Hong Kong West Cluster (Wave 1 reference number: UW 09‐387, approved on 4 November 2009; Wave 2 reference number: UW 11‐332, approved on 21 January 2013). Written parental consent was obtained for the surveyed adolescents. Approval covered data collection and data analysis for this study.

## Supporting information


**Appendix S1.** Supplemental text.
**Table S1.** STROBE Statement—Checklist of items that should be included in reports of cohort studies.
**Table S2.** Components and measures of positive self‐evaluation and environments.
**Table S3.** Life Event Checklist.
**Table S4.** Model fit statistics for associations between baseline exposures and behavioural well‐being at Wave 2 using Poisson regression – complete case analysis.
**Table S5.** Model fit statistics for associations between baseline exposures and emotional well‐being at Wave 2 using negative binomial regression – complete case analysis.
**Table S6.** Longitudinal Associations between baseline exposures and behavioural well‐being at Wave 2 using Poisson regression – complete case analysis.
**Table S7.** Longitudinal Associations between baseline exposures and emotional well‐being at Wave 2 using negative binomial regression – complete case analysis.
**Table S8.** Interactions between positive and stressful experiences.
**Table S9.** A mediation analysis of baseline mental health in the associations between baseline experiences and mental health at Wave 2.
**Table S10.** Interaction between baseline mental health and positive and stressful experiences.
**Figure S1.** A directed acyclic graph illustrating the choice of covariates and analytic strategy.
**Figure S2.** Conceptual models showing the relationship between baseline positive factors, baseline mental health and mental health at Wave 2.
**Appendix S2.** Stata codes for the association and interaction analyses.

## Data Availability

To protect participants' privacy, deidentified individual participant data are available under restricted access, as approved by the Institutional Review Board. Data access is limited to the Institutional Review Board for ethics review purposes and to authorised researchers for noncommercial academic use. Researchers must submit a research proposal for collaboration, which will be reviewed by the FAMILY Cohort Research Committee based on scientific merits, ethical review and regulatory requirements. Access will be granted only after the proposal is approved, and a signed undertaking on data transfer and access is received. Limited access to the data is also permitted for auditing and validation of this study, which requires a letter of intent outlining the intended purposes of data use. Requests for data access will be processed within 1 month. For detailed information on submitting a request, please visit https://www.familycohort.sph.hku.hk/database.
