# Supplementary material for: Adolescent mental health in Hong Kong: a longitudinal analysis of positive self‐evaluation, contextual influences and adverse experiences (2009–2014)
Source: Child Adolesc Ment Health. 2026 Feb 18;31(4):264–74. doi: 10.1111/camh.70073 (PMC13668474; doi:10.1111/camh.70073)
Supplement: Supplementary file 1 [file CAMH-31-264-s001.docx]

# Adolescent Mental Health in Hong Kong: A Longitudinal Analysis of Positive Self-Evaluation, Contextual Influences, and Adverse Experiences (2009–2014)

# Supporting Information

[Appendix S1.Supplemental text	2](#_Toc86559)

[Table S1 STROBE Statement—Checklist of items that should be included in reports of cohort studies…………………………………………………………………………………………………………………………………4](#_Toc86560)

[Table S2 Components and measures of positive self-evaluation and environments	6](#_Toc86562)

[Table S3 Life Event Checklist	7](#_Toc86563)

[Table S4 Model fit statistics for associations between baseline exposures and behavioural well-](#_Toc86564)

[being at wave 2 using Poisson regression – complete case analysis	8](#_Toc86565)

[Table S5 Model fit statistics for associations between baseline exposures and emotional well-](#_Toc86566)

[being at wave 2 using negative binomial regression – complete case analysis	10](#_Toc86567)

[Table S6 Longitudinal Associations between baseline exposures and behavioural well-being at wave 2 using Poisson regression – complete case analysis	12](#_Toc86568)

[Table S7 Longitudinal Associations between baseline exposures and emotional well-being at](#_Toc86569)

[wave 2 using negative binomial regression – complete case analysis	13](#_Toc86570)

[Table S8 Interactions between positive and stressful experiences	15](#_Toc86571)

[Table S9 A mediation analysis of baseline mental health in the associations between baseline](#_Toc86572)

[experiences and mental health at wave 2	16](#_Toc86573)

[Table S10 Interaction between baseline mental health and positive and stressful experiences	17](#_Toc86574)

[Figure S1 A directed acyclic graph illustrating the choice of covariates and analytic strategy	18](#_Toc86575)

[Figure S2 Conceptual models showing the relationship between baseline positive factors,](#_Toc86576)

[baseline mental health, and mental health at wave 2	19](#_Toc86577)

Appendix S2. [Stata codes for the association and interaction analyses	20](#_Toc86578)

**Appendix S1. Supplemental text**

## Weighting strategies

Inverse probability weighting was used to account for potential attrition bias in a prospective cohort study. Censoring weights were defined as the inverse probability of participating in the study after wave 1 and estimated using logistic regression with sociodemographic characteristics at wave 1 (i.e., child age, child sex, parental education, parental marital status, parental employment status, monthly household income and housing type). We then applied post-stratification weighting using raking for the sample to be representative of the 2011 Population By-census of Hong Kong using available data, including child age, child sex, and housing type.

## Mediation analysis

We performed a causal mediation analysis to analyse the mediating role of baseline mental health in the associations between baseline experiences and mental health at wave 2 using the R package CMAverse (Shi et al., 2021). A regression-based approach was adopted, and natural direct and indirect effects were estimated through direct counterfactual imputation estimation. Exposures were analysed as binary variables in the mediation analysis. For instance, scores of self-evaluation, peer relationship, and school experience were converted into binary variables as follows to indicate the presence of positive environment: (1) "yes" for scores of 4 (somewhat good) and 5 (very good); (2) "no" for scores of 1 (very bad), 2 (somewhat bad), and 3 (neither good nor bad). Regarding scores of family cohesion and family support, we considered a mean score greater than the mid-point of the rating scale as indicative of high family cohesion or high family support. In the case of family cohesion, the rating scale ranges from 1 to 6, making the midpoint 3.5. Therefore, we considered a mean score greater than 4 as high family cohesion. Negative binomial regression was used for both the mediator and the outcome for emotional well-being, while Poisson regression was used for both the mediator and the outcome for behavioural well-being. In interpreting our results, assumptions included no unmeasured confounding between exposure and outcome, mediators and outcome, and exposure and mediators after adjusting for sociodemographics; no intermediate confounding between mediators and outcome induced by exposure; no interactions between exposure and mediators; no measurement error; correct parametric specification of the models; no interference, and causal consistency (VanderWeele & Vansteelandt, 2014).

# Table S1 STROBE Statement—Checklist of items that should be included in reports of

# *cohort studies*

|  | **Item No** | | **Recommendation** | **Page No** |
| --- | --- | --- | --- | --- |
| **Title and abstract** | 1 | | (*a*) Indicate the study’s design with a commonly used term in the title or the abstract | 1 |
|  |  |  | (*b*) Provide in the abstract an informative and balanced summary of what was done and what was found | 1 |
| **Introduction** |  | |  |  |
| Background/rationale | 2 | | Explain the scientific background and rationale for the investigation being reported | 5-6 |
| Objectives | 3 | | State specific objectives, including any prespecified hypotheses | 6-7 |
| **Methods** |  | |  |  |
| Study design | 4 | | Present key elements of study design early in the paper | 7 |
| Setting | 5 | | Describe the setting, locations, and relevant dates, including periods of recruitment, exposure, follow-up, and data collection | 7 |
| Participants | 6 | | (*a*) Give the eligibility criteria, and the sources and methods of selection of participants. Describe methods of follow-up | 7 |
|  |  |  | (*b*) For matched studies, give matching criteria and number of exposed and unexposed | NA |
| Variables | 7 | | Clearly define all outcomes, exposures, predictors, potential confounders, and effect modifiers. Give diagnostic criteria, if applicable | 8-12 |
| Data sources/ measurement | 8* | | For each variable of interest, give sources of data and details of methods of assessment (measurement). Describe comparability of assessment methods if there is more than one group | 8-12 |
| Bias | 9 | | Describe any efforts to address potential sources of bias | 13-  14 |
| Study size | 10 | | Explain how the study size was arrived at | 7 |
| Quantitative variables | 11 | | Explain how quantitative variables were handled in the analyses. If applicable, describe which groupings were chosen and why | 8-12 |
| Statistical methods | 12 | | (*a*) Describe all statistical methods, including those used to control for confounding | 12-  14 |
|  |  |  | (*b*) Describe any methods used to examine subgroups and interactions | 13 |
|  |  |  | (*c*) Explain how missing data were addressed | 14 |
|  |  |  | (*d*) If applicable, explain how loss to follow-up was addressed | 14 |
|  |  |  | (*e*) Describe any sensitivity analyses | 14 |
| **Results** |  | |  |  |
| Participants | | 13* | (a) Report numbers of individuals at each stage of study— eg numbers potentially eligible, examined for eligibility, confirmed eligible, included in the study, completing followup, and analysed | 7 |
|  |  |  | (b) Give reasons for non-participation at each stage | 7 |
|  |  |  | (c) Consider use of a flow diagram | 7 |
| Descriptive data | | 14* | (a) Give characteristics of study participants (eg demographic, clinical, social) and information on exposures and potential confounders | 14 |
|  |  |  | (b) Indicate number of participants with missing data for each variable of interest | 14 |
|  |  |  | (c) Summarise follow-up time (eg, average and total amount) | 14 |
| Outcome data | | 15* | Report numbers of outcome events or summary measures over time | 14 |
| Main results | | 16 | (*a*) Give unadjusted estimates and, if applicable, confounder-adjusted estimates and their precision (eg, 95% confidence interval). Make clear which confounders were adjusted for and why they were included | 15-  16 |
|  |  |  | (*b*) Report category boundaries when continuous variables were categorized | 15-  16 |
|  |  |  | (*c*) If relevant, consider translating estimates of relative risk into absolute risk for a meaningful time period | NA |
| Other analyses | | 17 | Report other analyses done—eg analyses of subgroups and interactions, and sensitivity analyses | 14-  16 |
| **Discussion** | |  |  |  |
| Key results | | 18 | Summarise key results with reference to study objectives | 16 |
| Limitations | | 19 | Discuss limitations of the study, taking into account sources of potential bias or imprecision. Discuss both direction and magnitude of any potential bias | 21-  22 |
| Interpretation | | 20 | Give a cautious overall interpretation of results considering objectives, limitations, multiplicity of analyses, results from similar studies, and other relevant evidence | 17-  22 |
| Generalisability | | 21 | Discuss the generalisability (external validity) of the study results | 19,  22 |
| **Other information** | |  |  |  |
| Funding | | 22 | Give the source of funding and the role of the funders for the present study and, if applicable, for the original study on which the present article is based | 23 |

*Give information separately for exposed and unexposed groups.

**Note:** An Explanation and Elaboration article discusses each checklist item and gives methodological background and published examples of transparent reporting. The STROBE checklist is best used in conjunction with this article (freely available on the Web sites of PLoS Medicine at http://www.plosmedicine.org/, Annals of Internal Medicine at http://www.annals.org/, and Epidemiology at http://www.epidem.com/). Information on the STROBE Initiative is available at http://www.strobe-statement.org.

# Table S2 Components and measures of positive self-evaluation and environments

| **Components** | **Question** | **Response** |
| --- | --- | --- |
| 1. Positive selfevaluation | How good or bad have you felt about yourself in the last four weeks? (self-report) | 1=Very bad  2=Somewhat bad  3=Neither good nor bad  4=Somewhat good  5=Very good |
| 2. High family cohesion | The 9-item cohesion subscale of the Family  Environment Scale (FES, self-report) | 6-point Likert scale from 1=strongly agree to 6=strongly disagree; reversecoded, higher score indicating higher cohesion |
| 3. High family support | The 5-item Family APGAR (self-report) | 0=hardly ever to 2=almost always |
| 4. Positive peer relationship | How was your friendship in the past four weeks? (self-report) | 1=Very bad  2=Somewhat bad  3=Neither good nor bad  4=Somewhat good  5=Very good |
| 5. Positive school experience | How was your school work in the past four weeks? (self-report) | 1=Very bad  2=Somewhat bad  3=Neither good nor bad  4=Somewhat good  5=Very good |
| 6. Neighbourhood cohesion | The 5-item Neighborhood Social Cohesion  Scale (parent-report) | 1=strongly agree to 5=strongly disagree, reverse-coded, higher score indicating higher cohesion |

# Table S3 Life Event Checklist

| **List of stressful events in the past 12 months** | **Yes % (N=2171)** |
| --- | --- |
| 1. Close relative or friend died | 16.3 |
| 2. Had an illness or accident requiring hospitalization | 9.7 |
| 3. Was a victim of violence (physically harmed by someone) | 8.1 |
| 4. Was arrested or in serious trouble with the law | 4.0 |
| 5. Family’s financial situation got worse | 13.6 |
| 6. Broke up with girl/boyfriend | 4.3 |
| 7. Got pregnant (or girl got pregnant) [12-14y] | 2.8 [N=1214] |
| 8. Left home or moved out of home | 3.3 |
| 9. Important possession stolen | 11.1 |
| 10. A sudden increase in homework | 38.6 |
| 11. A sudden decline in school performance | 39.4 |
| 12. More conflicts with friends | 20.5 |
| 13. Increased arguments with parents – more than in the usual family | 19.8 |
| 14. Parents separated/divorced | 5.7 |

# Table S4 Model fit statistics for associations between baseline exposures and behavioural

# well-being at wave 2 using Poisson regression – complete case analysis

| **Exposures** | **Model fit statistics** | **Behavioural well-being** | |
| --- | --- | --- | --- |
| Positive self-evaluation | *N* | 1992 | |
|  | *Log pseudolikelihood* | -7267.8 | |
|  | *Wald chi2(14)* | 94.64 | |
|  | *Prob > chi2* | <0.001 | |
|  | *Deviance goodness-of-fit test P value* | 0.030 | |
|  | *Pearson goodness-of-fit test P value* | 0.903 | |
| High family cohesion | *N* | 1992 | |
|  | *Log pseudolikelihood* | -7271.6 | |
|  | *Wald chi2(14)* | 97.49 | |
|  | *Prob > chi2* | <0.001 | |
|  | *Deviance goodness-of-fit test P value* | 0.023 | |
|  | *Pearson goodness-of-fit test P value* | 0.885 | |
| High family support | *N* | 1992 | |
|  | *Log pseudolikelihood* | -7273.6 | |
|  | *Wald chi2(14)* | 91.86 | |
|  | *Prob > chi2* | <0.001 | |
|  | *Deviance goodness-of-fit test P value* | 0.020 | |
|  | *Pearson goodness-of-fit test P value* | 0.875 | |
| Positive peer relationship | *N* | 1992 | |
|  | *Log pseudolikelihood* | -7272.4 | |
|  | *Wald chi2(14)* | 91.7 | |
|  | *Prob > chi2* | <0.001 | |
|  | *Deviance goodness-of-fit test P value* | 0.022 | |
|  | *Pearson goodness-of-fit test P value* | 0.881 | |
| Positive school experience | *N* | 1992 | |
|  | *Log pseudolikelihood* | -7264.8 | |
|  | *Wald chi2(14)* | 97.04 | |
|  | *Prob > chi2* | <0.001 | |
|  | *Deviance goodness-of-fit test P value* | 0.037 | |
|  | *Pearson goodness-of-fit test P value* | 0.919 | |
| Neighbourhood cohesion | *N* | 1991 | |
|  | *Log pseudolikelihood* | -7273.1 | |
|  | *Wald chi2(14)* | 85.02 | |
|  | *Prob > chi2* | <0.001 | |
|  | *Deviance goodness-of-fit test P value* | 0.017 | |
|  | *Pearson goodness-of-fit test P value* | | 0.863 |
| Stressful life events | *N* | | 1992 |
|  | *Log pseudolikelihood* | | -7275 |
|  | *Wald chi2(16)* | | 88.77 |
|  | *Prob > chi2* | | <0.001 |
|  | *Deviance goodness-of-fit test P value* | | 0.018 |
|  | *Pearson goodness-of-fit test P value* | | 0.866 |
| School bullying victimisation | *N* | | 1992 |
|  | *Log pseudolikelihood* | | -7273.4 |
|  | *Wald chi2(14)* | | 89.42 |
|  | *Prob > chi2* | | <0.001 |
|  | *Deviance goodness-of-fit test P value* | | 0.020 |
|  | *Pearson goodness-of-fit test P value* | | 0.875 |
| Physical fighting | *N* | | 1992 |
|  | *Log pseudolikelihood* | | -7274 |
|  | *Wald chi2(14)* | | 86.73 |
|  | *Prob > chi2* | | <0.001 |
|  | *Deviance goodness-of-fit test P value* | | 0.019 |
|  | *Pearson goodness-of-fit test P value* | | 0.872 |

Notes: Positive factors were analysed as continuous scores. Model adjusted for child age and sex, parental education, employment, marital status, housing type, household income and behavioural well-being variable at wave 1.

# Table S5 Model fit statistics for associations between baseline exposures and emotional

# well-being at wave 2 using negative binomial regression – complete case analysis

| **Exposures** |  | | **Model fit statistics** | **Emotional well-being** | |
| --- | --- | --- | --- | --- | --- |
| Positive self-evaluation |  | | *N* | 1992 | |
|  |  | | *Log pseudolikelihood* | -7864.4 | |
|  |  | | *Wald chi2(14)* | 97.31 | |
|  |  | | *Prob > chi2* | <0.001 | |
|  |  | | *Pseudo R^2^* | 0.009 | |
|  |  | | *alpha (95% CI)* | 1.00 (0.98, 1.03) | |
| High family cohesion |  | | *N* | 1992 | |
|  |  | | *Log pseudolikelihood* | -7873.2 | |
|  |  | | *Wald chi2(14)* | 84.22 | |
|  |  | | *Prob > chi2* | 0 | |
|  |  | | *Pseudo R^2^* | 0.007 | |
|  |  | | *alpha (95% CI)* | 1.00 (0.98, 1.03) | |
| High family support |  | | *N* | 1992 | |
|  |  | | *Log pseudolikelihood* | -7877.1 | |
|  |  | | *Wald chi2(14)* | 74.01 | |
|  |  | | *Prob > chi2* | <0.001 | |
|  |  | | *Pseudo R^2^* | 0.007 | |
|  |  | | *alpha (95% CI)* | 1.00 (0.98, 1.03) | |
| Positive peer relationship |  | | *N* | 1992 | |
|  |  | | *Log pseudolikelihood* | -7874.5 | |
|  |  | | *Wald chi2(14)* | 82.69 | |
|  |  | | *Prob > chi2* | <0.001 | |
|  |  | | *Pseudo R^2^* | 0.007 | |
|  |  | | *alpha (95% CI)* | 1.00 (0.98, 1.03) | |
| Positive school experience |  | | *N* | 1992 | |
|  |  | | *Log pseudolikelihood* | -7874.5 | |
|  |  | | *Wald chi2(14)* | 86.89 | |
|  |  | | *Prob > chi2* | <0.001 | |
|  |  | | *Pseudo R^2^* | 0.007 | |
|  |  | | *alpha (95% CI)* | 1.00 (0.98, 1.03) | |
| Neighbourhood cohesion |  | | *N* | 1991 | |
|  |  | | *Log pseudolikelihood* | -7875.7 | |
|  |  | | *Wald chi2(14)* | 72.13 | |
|  |  | | *Prob > chi2* | <0.001 | |
|  |  | | *Pseudo R^2^* | 0.007 | |
|  | |  | *alpha (95% CI)* | | 1.00 (0.98, 1.03) |
| Stressful life events | |  | *N* | | 1992 |
|  | |  | *Log pseudolikelihood* | | -7876.7 |
|  | |  | *Wald chi2(16)* | | 77.94 |
|  | |  | *Prob > chi2* | | <0.001 |
|  | |  | *Pseudo R^2^* | | 0.007 |
|  | |  | *alpha (95% CI)* | | 1.00 (0.98, 1.03) |
| School bullying victimisation | |  | *N* | | 1992 |
|  | |  | *Log pseudolikelihood* | | -7877.8 |
|  | |  | *Wald chi2(14)* | | 73.39 |
|  | |  | *Prob > chi2* | | <0.001 |
|  | |  | *Pseudo R^2^* | | 0.007 |
|  | |  | *alpha (95% CI)* | | 1.00 (0.98, 1.03) |
| Physical fighting | |  | *N* | | 1992 |
|  | |  | *Log pseudolikelihood* | | -7876.9 |
|  | |  | *Wald chi2(14)* | | 78.01 |
|  | |  | *Prob > chi2* | | <0.001 |
|  | |  | *Pseudo R^2^* | | 0.007 |
|  | |  | *alpha (95% CI)* | | 1.00 (0.98, 1.03) |

Notes: Positive factors were analysed as continuous scores. Model adjusted for child age and sex, parental education, employment, marital status, housing type, household income and emotional well-being variable at wave 1.

# Table S6 Longitudinal Associations between baseline exposures and behavioural wellbeing at wave 2 using Poisson regression – complete case analysis

|  | **Model 1 IRR (95% CI)** | **Model 2 IRR (95% CI)** | **Model 3 IRR (95% CI)** |
| --- | --- | --- | --- |
| **Positive factor scores**  Positive self-evaluation | **1.03 (1.02, 1.04)**  **[N=2171]** | **1.03 (1.02, 1.04)**  **[N=1992]** | **1.01 (1.00, 1.02)**  **[N=1992] (p=0.006)** |
| High family cohesion | **1.02 (1.01, 1.03)**  **[N=2171]** | **1.02 (1.01, 1.03)**  **[N=1992]** | **1.01 (1.00, 1.02) [N=1992] (p=0.032)** |
| High family support | **1.02 (1.01, 1.03)**  **[N=2171]** | **1.02 (1.01, 1.03)**  **[N=1992]** | 1.01 (1.00, 1.02)  [N=1992] |
| Positive peer relationship | **1.02 (1.01, 1.02)**  **[N=2171]** | **1.02 (1.01, 1.02)**  **[N=1992]** | **1.01 (1.00, 1.02) [N=1992] (p=0.046)** |
| Positive school experience | **1.03 (1.02, 1.03)**  **[N=2171]** | **1.02 (1.02, 1.03)**  **[N=1992]** | **1.01 (1.01, 1.02)**  **[N=1992]** |
| Neighbourhood cohesion | 1.00 (0.99, 1.01)  [N=2117] | 1.00 (0.99, 1.01)  [N=1991] | 1.00 (0.99, 1.01)  [N=1991] |
| **Stressful life events**  (ref.=no)  1 event | 1.00 (0.99, 1.02)  [N=2171] | 1.00 (0.99, 1.02)  [N=1992] | 1.00 (0.99, 1.02)  [N=1992] |
| 2 events | 1.00 (0.98, 1.01)  [N=2171] | 1.00 (0.98, 1.01)  [N=1992] | 1.00 (0.99, 1.02)  [N=1992] |
| ≥3 events | **0.97 (0.96, 0.99)**  **[N=2171]** | **0.98 (0.96, 0.99)**  **[N=1992]** | 1.00 (0.99, 1.01)  [N=1992] |
| **School bullying victimisation**  (ref.=no) | **0.96 (0.94, 0.98)**  **[N=2171]** | **0.96 (0.94, 0.98)**  **[N=1992]** | 0.98 (0.96, 1.01)  [N=1992] |
| **Physical fighting**  (ref.=no) | **0.98 (0.97, 0.99)**  **[N=2171]** | **0.98 (0.96, 0.99)**  **[N=1992]** | 0.99 (0.98, 1.01)  [N=1992] |

Notes: IRR, incidence rate ratio. Positive factors were analysed as continuous scores. Model 1: unadjusted. Model 2: adjusted for child age and sex, parental education, employment, marital status, housing type and household income at wave 1. Model 3: further adjusted for behavioural well-being at wave 1. Bold fonts indicate significant associations.

# Table S7 Longitudinal Associations between baseline exposures and emotional well-being at

# wave 2 using negative binomial regression – complete case analysis

|  | **Model 1 IRR (95% CI)** | **Model 2 IRR (95% CI)** |  | **Model 3 IRR (95% CI)** |
| --- | --- | --- | --- | --- |
| **Positive factor scores**  Positive self-evaluation | **1.04 (1.03, 1.05)**  **[N=2171]** | **1.04 (1.03, 1.05)**  **[N=1992]** |  | **1.02 (1.01, 1.03)**  **[N=1992]** |
| High family cohesion | **1.03 (1.02, 1.04)**  **[N=2171]** | **1.03 (1.02, 1.04)**  **[N=1992]** |  | 1.01 (0.99, 1.02)  [N=1992] |
| High family support | **1.03 (1.01, 1.05)**  **[N=2171]** | **1.02 (1.01, 1.04)**  **[N=1992]** |  | 0.99 (0.98, 1.01)  [N=1992] |
| Positive peer relationship | **1.02 (1.01, 1.04)**  **[N=2171]** | **1.02 (1.01, 1.03)**  **[N=1992]** |  | 1.01 (1.00, 1.02)  [N=1992] |
| Positive school experience | **1.03 (1.02, 1.04)**  **[N=2171]** | **1.02 (1.01, 1.03)**  **[N=1992]** |  | 1.01 (1.00, 1.02) [N=1992] (p=0.085) |
| Neighbourhood cohesion | 1.01 (0.99, 1.02)  [N=2117] | 1.01 (0.99, 1.02)  [N=1991] |  | 1.00 (0.99, 1.01)  [N=1991] |
| **Stressful life events**  (ref.=no)  1 event | 0.99 (0.97, 1.02)  [N=2171] | 1.00 (0.98, 1.02)  [N=1992] |  | 1.00 (0.98, 1.02)  [N=1992] |
| 2 events | **0.97 (0.95, 1.00)**  **[N=2171]** | **0.97 (0.95, 1.00)**  **[N=1992]** |  | 0.99 (0.97, 1.01)  [N=1992] |
| ≥3 events | **0.96 (0.94, 0.98)**  **[N=2171]** | **0.97 (0.95, 0.99)**  **[N=1992]** |  | 1.00 (0.98, 1.02)  [N=1992] |
| **School bullying victimisation**  (ref.=no) | 0.97 (0.94, 1.01)  [N=2171] | 0.97 (0.94, 1.00)  [N=1992] |  | 1.00 (0.97, 1.03)  [N=1992] |
| **Physical fighting**  (ref.=no) | 0.99 (0.97, 1.01)  [N=2171] | **0.97 (0.95, 0.99)**  **[N=1992]** |  | 0.99 (0.97, 1.00)  [N=1992] |

Notes: IRR, incidence rate ratio. Positive factors were analysed as continuous scores. Model 1: unadjusted. Model 2: adjusted for child age and sex, parental education, employment, marital status, housing type and household income at wave 1. Model 3: further adjusted for emotional well-being at wave 1. Bold fonts indicate p values <0.05.

# Table S8 Interactions between positive and stressful experiences

|  | **Behavioural well-being**  ***p* value** | **Emotional well-being**  ***p* value** |
| --- | --- | --- |
| **Interactions with life stress** | **[N=2171]** | **[N=2171]** |
| Positive self-evaluation*life stress | 0.074 | 0.661 |
| High family cohesion*life stress | 0.725 | 0.111 |
| High family support*life stress | 0.845 | 0.207 |
| Positive peer relationship*life stress | 0.009 | 0.38 |
| Positive school experience*life stress | 0.773 | 0.641 |
| Neighbourhood cohesion*life stress | 0.823 | 0.536 |
| **Interactions with school bullying victimisation**  Positive self-evaluation*bullying victimisation | 0.254 | 0.373 |
| High family cohesion*bullying victimisation | 0.399 | 0.597 |
| High family support*bullying victimisation | 0.792 | 0.55 |
| Positive peer relationship*bullying victimisation | 0.600 | 0.474 |
| Positive school experience*bullying victimisation | 0.319 | 0.207 |
| Neighbourhood cohesion*bullying victimisation | 0.759 | 0.653 |
| **Interactions with physical fighting**  Positive self-evaluation*physical fighting | 0.814 | 0.791 |
| High family cohesion*physical fighting | 0.892 | 0.954 |
| High family support*physical fighting | 0.221 | 0.811 |
| Positive peer relationship*physical fighting | 0.903 | 0.358 |
| Positive school experience*physical fighting | 0.71 | 0.384 |
| Neighbourhood cohesion*physical fighting | 0.055 | 0.210 |

Notes: Positive factors were analysed as continuous scores. Interactions from fully adjusted models and results with multiple imputations for missing data are presented. Following the association analysis, life stress was further categorised into binary variables for the interaction analysis: (1) behavioural well-being: <3 versus ≥3 events; (2) emotional well-being: <2 versus ≥2 events.

# Table S9 A mediation analysis of baseline mental health in the associations between baseline

# experiences and mental health at wave 2

|  | **NDE** | **TNIE** | **TE** | **Proportion mediated** |
| --- | --- | --- | --- | --- |
| **Behavioural well-being** | **Estimate (95% CI)** | **Estimate (95%**  **CI)** | **Estimate (95%**  **CI)** | **% (95% CI)** |
| Positive self-evaluation | 1.01 (1.00, 1.02)  [*p*=0.06] | **1.02 (1.01, 1.02)** | **1.03 (1.02, 1.04)** | **56.2 (41.8, 100)** |
| High family cohesion | 1.01 (1.00, 1.03) | **1.02 (1.01, 1.03)** | **1.03 (1.02, 1.05)** | **58.7 (39.9, 100)** |
| High family support | 1.01 (1.00, 1.02) | **1.01 (1.01, 1.02)** | **1.02 (1.01, 1.03)** | **55.9 (37.2, 100)** |
| Positive peer relationship | 1.01 (1.00, 1.03) | **1.01 (1.01, 1.02)** | **1.03 (1.01, 1.04)** | **48.8 (34.1, 100)** |
| Positive school experience | **1.02 (1.00, 1.02)** | **1.01 (1.01, 1.02)** | **1.03 (1.02, 1.04)** | **48.6 (32.1, 80.1)** |
| Life stress | 1.00 (0.98, 1.00) | **0.98 (0.97, 0.98)** | **0.97 (0.96, 0.98)** | **85.3 (47.5, 100)** |
| Bullying victimisation | 0.99 (0.96, 1.01) | **0.98 (0.96, 0.98)** | **0.96 (0.93, 0.98)** | **61.7 (36.8, 100)** |
| Physical fighting | 0.99 (0.98, 1.00) | **0.98 (0.98, 0.99)** | **0.98 (0.96, 0.99)** | **74.3 (36.0, 100)** |
| **Emotional well-being**  Positive self-evaluation | **1.02 (1.01, 1.04)** | **1.03 (1.02, 1.03)** | **1.05 (1.03, 1.06)** | **53.2 (36.4, 79.4)** |
| High family cohesion | 1.00 (0.98, 1.02) | 1.03 (1.02, 1.04) | 1.04 (1.01, 1.05) | **86.7 (52.2, 100)** |
| High family support | 1.00 (0.98, 1.01) | 1.03 (1.02, 1.03) | 1.02 (1.01, 1.04) | **119.7 (67.3, 100)** |
| Positive peer relationship | 1.02 (1.00, 1.04)  [*p*=0.05] | 1.03 (1.02, 1.03) | 1.05 (1.02, 1.06) | **54.9 (39.1, 98.0)** |
| Positive school experience | 1.01 (1.00, 1.03)  [*p*=0.06] | 1.02 (1.01, 1.02) | 1.03 (1.01, 1.05) | **60.1 (36.2, 100)** |
| Life stress | 1.01 (0.99, 1.02) | 0.96 (0.96, 0.98) | 0.97 (0.95, 0.99) | - |
| Bullying victimisation | 1.00 (0.96, 1.02) | 0.97 (0.96, 0.98) | 0.96 (0.92, 0.99) | **86.8 (41.7, 100)** |
| Physical fighting | 0.99 (0.97, 1.01) | 0.99 (0.98, 0.99) | 0.98 (0.96, 1.00) | **67.9 (29.5, 100)** |

Notes: NDE, natural direct effect rate ratio; TNIE, total natural indirect effect rate ratio; TE, total effect rate ratio. Models adjusted for child age and sex, parental education, employment, marital status, housing type and household income at wave 1. Imputed results are presented. Bold fonts indicate *p* values <0.05. We did not report the proportion mediated by baseline emotional well-being in the association between life stress and emotional well-being at wave 2 given the number exceeding 100%.

# Table S10 Interaction between baseline mental health and positive and stressful experiences

|  | **Behavioural well-being**  ***p* value** | **Emotional well-being**  ***p* value** |
| --- | --- | --- |
|  | **[N=2171]** | **[N=2171]** |
| Positive self-evaluation*baseline mental health | 0.149 | 0.393 |
| High family cohesion* baseline mental health | 0.625 | 0.057 |
| High family support* baseline mental health | 0.577 | 0.192 |
| Positive peer relationship* baseline mental health | 0.518 | 0.088 |
| Positive school experience*baseline mental health | 0.269 | 0.272 |
| Neighbourhood cohesion*baseline mental health | 0.604 | 0.634 |

Notes: Positive factors were analysed as continuous scores. Models adjusted for child age and sex, parental education, employment, marital status, housing type and household income at wave 1. Results with multiple imputations for missing data are presented. Following the association analysis, life stress was further categorised into binary variables for the interaction analysis: (1) behavioural well-being: <3 versus ≥3 events; (2) emotional well-being: <2 versus ≥2 events.

# Figure S1 A directed acyclic graph illustrating the choice of covariates and analytic strategy

Housing type

and household

income

at

baseline

Baseline

p

ositive or

adverse

experiences

Child age

and sex

at

baseline

Parental

education,

employment, and

marital status

at

baseline

Adolescent

mental

health at

wave 2

Baseline

mental

health

Confounder

Confounder

Confounder

Confounder

Exposure

Outcome

# Figure S2 Conceptual models showing the relationship between baseline positive factors,

# baseline mental health, and mental health at wave 2

Positive factors

at

baseline

Mental health at

baseline

Adolescent mental

health at wave 2

Positive factors ×

mental

health at baseline

2

2

1

Notes: Positive factors included positive self-evaluation and positive environments created by family, peers and schools, and neighbourhoods. Full models adjusted for baseline covariates, including child age and sex, parental education, employment, marital status, housing type and household income.

# Appendix S2. Stata codes for the association and interaction analyses

******Codes for the outcome of emotional wellbeing at wave 2**

### *Prepare data for multiple imputation

use youthdataWave12_weights_method2.dta, clear

keep crid rhid weights chqcf87mh_w2 chqcf87mh_w1 lse_cat4 chyrbsbb_w1 ph_w1 chsex_w1 chage_w1 pedulevel pemply_a pmar_b htype2_2 hhincome_2nsgp cf87ca711_w1 m_fco m_ffu cf87ca714_w1 cf87ca712_w1 m_nc keep if !missing(weights) recode pmar_b (1=1) (2=0)

*Let's summarise missing values misstable summarize, generate(Mis_)

*and explore missing-data patterns misstable patterns

*Declare the storage style mi set mlong

*Register variables mi register imputed hhincome_2nsgp pedulevel htype2_2 pemply_a pmar_b m_nc

mi register regular weights chqcf87mh_w1 chqcf87mh_w2 chsex_w1 chage_w1 cf87ca711_w1 m_fco m_ffu cf87ca714_w1 cf87ca712_w1 lse_cat4 chyrbsbb_w1 ph_w1

*multiple imputation mi impute chained (reg) m_nc (mlogit) hhincome_2nsgp (mlogit) pedulevel htype2_2 pemply_a /// (logit) pmar_b ///

= chqcf87mh_w1 chqcf87mh_w2 i.chsex_w1 chage_w1 cf87ca711_w1 m_fco m_ffu cf87ca714_w1 cf87ca712_w1 i.lse_cat4 i.chyrbsbb_w1 i.ph_w1 [pweight = weights], /// add(20) burnin(10) savetrace(impstats_e1, replace) rseed(27654) noisily augment

****

mi passive: gen lse_cat2e=0 if lse_cat4==0 | lse_cat4==1 mi passive: replace lse_cat2e=1 if lse_cat4==3 | lse_cat4==2 save ymh_mi_mh_con.dta,replace

*****Model estimates** use ymh_mi_mh_con.dta,clear

**MI Table 1 for binary exposures local myoutlist mh foreach myo in `myoutlist' {

tempname myhandle

file open `myhandle' using ymh_table_`myo'_mi_nb_con.txt, write replace file write `myhandle' "MI Table 1 Associations between positive/adverse experiences and outcomes at wave 2" _n file write `myhandle' _tab ("Model1 B (95%)") _tab ("Model2 B (95%)") _tab ("p1") _tab ("p2") _n

local myvlist cf87ca711_w1 m_fco m_ffu cf87ca714_w1 cf87ca712_w1 m_nc chyrbsbb_w1 ph_w1 local myclist1 i.chsex_w1 chage_w1 i.pedulevel i.pemply_a i.pmar_b i.htype2_2 i.hhincome_2nsgp

local myclist2 i.chsex_w1 chage_w1 chqcf87`myo'_w1 i.pedulevel i.pemply_a i.pmar_b i.htype2_2 i.hhincome_2nsgp

file write `myhandle' "chqcf87`myo'" _n

foreach myv in `myvlist' {	file write `myhandle' "`myv'"

mi estimate, irr cmdok: nbreg chqcf87`myo'_w2 `myv' [pweight = weights], vce(cl rhid)

matrix define myresults=r(table)	local myn: display %3.0f e(N)	local mymd: display %5.2f myresults[1,1]	local mypvalue1: display %5.3f myresults[4,1]	local mylci: display %5.2f myresults[5,1]	local myuci: display %5.2f myresults[6,1]	local mci="`mymd'" + " (" + "`mylci'"+ ", " + "`myuci'" + ")" + " [N=" + "`myn'" + "]"	file write `myhandle' _tab ("`mci'")

| rhid) |  | mi estimate, irr cmdok: nbreg chqcf87`myo'_w2 `myv' `myclist1' [pweight = weights], vce(cl |
| --- | --- | --- |
|  |  | matrix define myresults=r(table) |
|  |  | local myn: display %3.0f e(N) |
|  |  | local mymd: display %5.2f myresults[1,1] |
|  |  | local mypvalue2: display %5.3f myresults[4,1] |
|  |  | local mylci: display %5.2f myresults[5,1] |
|  |  | local myuci: display %5.2f myresults[6,1] |
|  |  | local mci="`mymd'" + " (" + "`mylci'"+ ", " + "`myuci'" + ")" + " [N=" + "`myn'" + "]" |
|  |  | file write `myhandle' _tab ("`mci'") |
|  |  |  |
| rhid) |  | mi estimate, irr cmdok: nbreg chqcf87`myo'_w2 `myv' `myclist2' [pweight = weights], vce(cl |
|  |  | matrix define myresults=r(table) |
|  |  | local myn: display %3.0f e(N) |
|  |  | local mymd: display %5.2f myresults[1,1] |
|  |  | local mypvalue3: display %5.3f myresults[4,1] |
|  |  | local mylci: display %5.2f myresults[5,1] |
|  |  | local myuci: display %5.2f myresults[6,1] |
|  |  | local mci="`mymd'" + " (" + "`mylci'"+ ", " + "`myuci'" + ")" + " [N=" + "`myn'" + "]" |
|  |  | file write `myhandle' _tab ("`mci'") |
|  |  | file write `myhandle' _tab ("`mypvalue1'") _tab ("`mypvalue2'") _tab ("`mypvalue3'") |

file write `myhandle' _n

}/*close of the myvar loop*/

file close `myhandle' di as txt `"(Results are in {browse "ymh_table_`myo'_mi_nb_con.txt"})"' }/*close of the myout loop*/

*MI Table2 lse_cat4 local myoutlist mh foreach myo in `myoutlist' { tempname myhandle file open `myhandle' using ymh_table2_`myo'_lse_cat4_mi_nb_con.txt, write replace file write `myhandle' "Table 2 Associations between stressful experiences and outcomes at wave 2" _n local myvlist lse_cat4 local myclist1 i.chsex_w1 chage_w1 i.pedulevel i.pemply_a i.pmar_b i.htype2_2 i.hhincome_2nsgp

local myclist2 i.chsex_w1 chage_w1 chqcf87`myo'_w1 i.pedulevel i.pemply_a i.pmar_b i.htype2_2 i.hhincome_2nsgp file write `myhandle' "chqcf87`myo'" _n

foreach myv in `myvlist' {	file write `myhandle' "`myv'"

mi estimate, irr cmdok: nbreg chqcf87`myo'_w2 i.`myv' [pweight = weights], vce(cl rhid)

matrix define myresults=r(table)	local myn: display %3.0f e(N)	local mymd: display %5.2f myresults[1,2]	local mypvalue: display %5.3f myresults[4,2]	local mylci: display %5.2f myresults[5,2]	local myuci: display %5.2f myresults[6,2]	local mci="`mymd'" + " (" + "`mylci'"+ ", " + "`myuci'" + ")" + " [N=" + "`myn'" + "]"	file write `myhandle' _tab ("`mci'") _tab ("`mypvalue'") _n

local myn: display %3.0f e(N)	local mymd: display %5.2f myresults[1,3]	local mypvalue: display %5.3f myresults[4,3]	local mylci: display %5.2f myresults[5,3]	local myuci: display %5.2f myresults[6,3]	local mci="`mymd'" + " (" + "`mylci'"+ ", " + "`myuci'" + ")" + " [N=" + "`myn'" + "]"	file write `myhandle' _tab ("`mci'") _tab ("`mypvalue'") _n

local myn: display %3.0f e(N)	local mymd: display %5.2f myresults[1,4]	local mypvalue: display %5.3f myresults[4,4]	local mylci: display %5.2f myresults[5,4]	local myuci: display %5.2f myresults[6,4]	local mci="`mymd'" + " (" + "`mylci'"+ ", " + "`myuci'" + ")" + " [N=" + "`myn'" + "]"	file write `myhandle' _tab ("`mci'") _tab ("`mypvalue'") _n

| rhid) |  | mi estimate, irr cmdok: nbreg chqcf87`myo'_w2 i.`myv' `myclist1' [pweight = weights], vce(cl |
| --- | --- | --- |
|  |  | matrix define myresults=r(table) |
|  |  | local myn: display %3.0f e(N) |
|  |  | local mymd: display %5.2f myresults[1,2] |
|  |  | local mypvalue: display %5.3f myresults[4,2] |
|  |  | local mylci: display %5.2f myresults[5,2] |
|  |  | local myuci: display %5.2f myresults[6,2] |
|  |  | local mci="`mymd'" + " (" + "`mylci'"+ ", " + "`myuci'" + ")" + " [N=" + "`myn'" + "]" |
|  |  | file write `myhandle' _tab ("`mci'") _tab ("`mypvalue'") _n |
|  |  |  |
|  |  | local myn: display %3.0f e(N) |
|  |  | local mymd: display %5.2f myresults[1,3] |
|  |  | local mypvalue: display %5.3f myresults[4,3] |
|  |  | local mylci: display %5.2f myresults[5,3] |
|  |  | local myuci: display %5.2f myresults[6,3] |
|  |  | local mci="`mymd'" + " (" + "`mylci'"+ ", " + "`myuci'" + ")" + " [N=" + "`myn'" + "]" |
|  |  | file write `myhandle' _tab ("`mci'") _tab ("`mypvalue'") _n |
|  |  |  |
|  |  | local myn: display %3.0f e(N) |
|  |  | local mymd: display %5.2f myresults[1,4] |
|  |  | local mypvalue: display %5.3f myresults[4,4] |
|  |  | local mylci: display %5.2f myresults[5,4] |
|  |  | local myuci: display %5.2f myresults[6,4] |
|  |  | local mci="`mymd'" + " (" + "`mylci'"+ ", " + "`myuci'" + ")" + " [N=" + "`myn'" + "]" |
|  |  | file write `myhandle' _tab ("`mci'") _tab ("`mypvalue'") _n |
|  |  |  |
| rhid) |  | mi estimate, irr cmdok: nbreg chqcf87`myo'_w2 i.`myv' `myclist2' [pweight = weights], vce(cl |
|  |  | matrix define myresults=r(table) |
|  |  | local myn: display %3.0f e(N) |
|  |  | local mymd: display %5.2f myresults[1,2] |
|  |  | local mypvalue: display %5.3f myresults[4,2] |

local mylci: display %5.2f myresults[5,2]	local myuci: display %5.2f myresults[6,2]	local mci="`mymd'" + " (" + "`mylci'"+ ", " + "`myuci'" + ")" + " [N=" + "`myn'" + "]"	file write `myhandle' _tab ("`mci'") _tab ("`mypvalue'") _n

local myn: display %3.0f e(N)

local mymd: display %5.2f myresults[1,3]	local mypvalue: display %5.3f myresults[4,3]	local mylci: display %5.2f myresults[5,3]	local myuci: display %5.2f myresults[6,3]	local mci="`mymd'" + " (" + "`mylci'"+ ", " + "`myuci'" + ")" + " [N=" + "`myn'" + "]"	file write `myhandle' _tab ("`mci'") _tab ("`mypvalue'") _n

local myn: display %3.0f e(N)

local mymd: display %5.2f myresults[1,4]	local mypvalue: display %5.3f myresults[4,4]	local mylci: display %5.2f myresults[5,4]	local myuci: display %5.2f myresults[6,4]	local mci="`mymd'" + " (" + "`mylci'"+ ", " + "`myuci'" + ")" + " [N=" + "`myn'" + "]"	file write `myhandle' _tab ("`mci'") _tab ("`mypvalue'")	file write `myhandle' _n	}/*close of the myvar loop*/

file close `myhandle' di as txt `"(Results are in {browse "ymh_table2_`myo'_lse_cat4_mi_nb_con.txt"})"'	}/*close of the myout loop*/

**MI model for interaction use ymh_mi_mh_con.dta,clear tempname myhandle file open `myhandle' using ymh_table11a_mi_mh_nb_con.txt, write replace file write `myhandle' "Table 11a Interactions between positive and stressful experiences using nb regression" _n

file write `myhandle' _tab ("Model1 B (95%)") _tab ("Model2 B (95%)") _tab ("Model3 B (95%)") _tab ("p1") _tab ("p2") _tab ("p3") _n

local myoutlist chqcf87mh_w2 local myvlist2 cf87ca711_w1 m_fco m_ffu cf87ca714_w1 cf87ca712_w1 m_nc local myvlist lse_cat2e chyrbsbb_w1 ph_w1 local myclist1 i.chsex_w1 chage_w1 i.pedulevel i.pemply_a i.pmar_b i.htype2_2 i.hhincome_2nsgp local myclist2 i.chsex_w1 chage_w1 chqcf87mh_w1 i.pedulevel i.pemply_a i.pmar_b i.htype2_2 i.hhincome_2nsgp

foreach myo in `myoutlist' {	file write `myhandle' "`myo'" _n	foreach myv in `myvlist' {	foreach myv2 in `myvlist2' {

*file write `myhandle' "`myv'" _n

mi estimate, irr cmdok: nbreg `myo' i.`myv'##c.`myv2' [pweight = weights],vce(cl rhid)

matrix define myresults=r(table)	local myn1: display %3.0f e(N)	local mymd1: display %5.2f myresults[1,2]	local mypvalue1: display %5.3f myresults[4,2]	local mylci1: display %5.2f myresults[5,2]	local myuci1: display %5.2f myresults[6,2]	local mci1="`mymd1'" + " (" + "`mylci1'"+ ", " + "`myuci1'" + ")"

local myn2: display %3.0f e(N)	local mymd2: display %5.2f myresults[1,3]	local mypvalue2: display %5.3f myresults[4,3]	local mylci2: display %5.2f myresults[5,3]	local myuci2: display %5.2f myresults[6,3]	local mci2="`mymd2'" + " (" + "`mylci2'"+ ", " + "`myuci2'" + ")"

local myn3: display %3.0f e(N)	local mymd3: display %5.2f myresults[1,5]	local mypvalue3: display %5.3f myresults[4,5]	local mylci3: display %5.2f myresults[5,5]	local myuci3: display %5.2f myresults[6,5]	local mci3="`mymd3'" + " (" + "`mylci3'"+ ", " + "`myuci3'" + ")"

| rhid) |  | mi estimate, irr cmdok: nbreg `myo' i.`myv'##c.`myv2' `myclist1' [pweight = weights], vce(cl |
| --- | --- | --- |
|  |  | matrix define myresults=r(table) |
|  |  | local myn4: display %3.0f e(N) |
|  |  | local mymd4: display %5.2f myresults[1,2] |
|  |  | local mypvalue4: display %5.3f myresults[4,2] |
|  |  | local mylci4: display %5.2f myresults[5,2] |
|  |  | local myuci4: display %5.2f myresults[6,2] |
|  |  | local mci4="`mymd4'" + " (" + "`mylci4'"+ ", " + "`myuci4'" + ")" |
|  |  |  |
|  |  | local myn5: display %3.0f e(N) |
|  |  | local mymd5: display %5.2f myresults[1,3] |
|  |  | local mypvalue5: display %5.3f myresults[4,3] |
|  |  | local mylci5: display %5.2f myresults[5,3] |
|  |  | local myuci5: display %5.2f myresults[6,3] |
|  |  | local mci5="`mymd5'" + " (" + "`mylci5'"+ ", " + "`myuci5'" + ")" |
|  |  |  |
|  |  | local myn6: display %3.0f e(N) |
|  |  | local mymd6: display %5.2f myresults[1,5] |
|  |  | local mypvalue6: display %5.3f myresults[4,5] |
|  |  | local mylci6: display %5.2f myresults[5,5] |
|  |  | local myuci6: display %5.2f myresults[6,5] |
|  |  | local mci6="`mymd6'" + " (" + "`mylci6'"+ ", " + "`myuci6'" + ")" |
|  |  |  |
| rhid) |  | mi estimate, irr cmdok: nbreg `myo' i.`myv'##c.`myv2' `myclist2' [pweight = weights], vce(cl |
|  |  | matrix define myresults=r(table) |
|  |  | local myn7: display %3.0f e(N) |
|  |  | local mymd7: display %5.2f myresults[1,2] |
|  |  | local mypvalue7: display %5.3f myresults[4,2] |
|  |  | local mylci7: display %5.2f myresults[5,2] |
|  |  | local myuci7: display %5.2f myresults[6,2] |
|  |  | local mci7="`mymd7'" + " (" + "`mylci7'"+ ", " + "`myuci7'" + ")" |
|  |  |  |
|  |  | local myn8: display %3.0f e(N) |

local mymd8: display %5.2f myresults[1,3]	local mypvalue8: display %5.3f myresults[4,3]	local mylci8: display %5.2f myresults[5,3]	local myuci8: display %5.2f myresults[6,3]	local mci8="`mymd8'" + " (" + "`mylci8'"+ ", " + "`myuci8'" + ")"

local myn9: display %3.0f e(N)	local mymd9: display %5.2f myresults[1,5]	local mypvalue9: display %5.3f myresults[4,5]	local mylci9: display %5.2f myresults[5,5]	local myuci9: display %5.2f myresults[6,5]	local mci9="`mymd9'" + " (" + "`mylci9'"+ ", " + "`myuci9'" + ")"

*file write `myhandle' "`myv'" _tab ("`mci1'") _tab ("`mci4'") _tab ("`mci7'") _tab ("`mypvalue1'") _tab ("`mypvalue4'") _tab ("`mypvalue7'") _n

*file write `myhandle' "`myv2'" _tab ("`mci2'") _tab ("`mci5'") _tab ("`mci8'") _tab ("`mypvalue2'")

_tab ("`mypvalue5'") _tab ("`mypvalue8'") _n

file write `myhandle' "`myv'*`myv2'" _tab ("`mci3'") _tab ("`mci6'") _tab ("`mci9'") _tab ("`mypvalue3'") _tab ("`mypvalue6'") _tab ("`mypvalue9'") _n

*file write `myhandle' _n

}/*close of the myvar loop*/

}/*close of the myvar loop*/

}/*close of the myout loop*/ file close `myhandle' di as txt `"(Results are in {browse "ymh_table11a_mi_mh_nb_con.txt"})"'

### ****Codes for the outcome of behavioural wellbeing at wave 2

***Prepare data for multiple imputation** use youthdataWave12_weights_method2.dta, clear

keep crid rhid weights chqcf87be_w2 chqcf87be_w1 lse_cat4 chyrbsbb_w1 ph_w1 chsex_w1 chage_w1 pedulevel pemply_a pmar_b htype2_2 hhincome_2nsgp cf87ca711_w1 m_fco m_ffu cf87ca714_w1 cf87ca712_w1 m_nc keep if !missing(weights) recode pmar_b (1=1) (2=0)

*Let's summarise missing values misstable summarize, generate(Mis_)

*and explore missing-data patterns misstable patterns

*Declare the storage style mi set mlong

*Register variables mi register imputed hhincome_2nsgp pedulevel htype2_2 pemply_a pmar_b m_nc

mi register regular weights chqcf87be_w1 chqcf87be_w2 chsex_w1 chage_w1 cf87ca711_w1 m_fco m_ffu cf87ca714_w1 cf87ca712_w1 lse_cat4 chyrbsbb_w1 ph_w1

*Multiple imputation mi impute chained (reg) m_nc (mlogit) hhincome_2nsgp (mlogit) pedulevel htype2_2 pemply_a /// (logit) pmar_b ///

= chqcf87be_w1 chqcf87be_w2 i.chsex_w1 chage_w1 cf87ca711_w1 m_fco m_ffu cf87ca714_w1 cf87ca712_w1 i.lse_cat4 i.chyrbsbb_w1 i.ph_w1 [pweight = weights], /// add(20) burnin(10) savetrace(impstats_e1, replace) rseed(27654) noisily augment

****MI passive	mi passive: gen lse_cat2e=0 if lse_cat4==0 | lse_cat4==1 | lse_cat4==2	mi passive: replace lse_cat2e=1 if lse_cat4==3

mi passive: gen pce5=0 if cf87ca714_w1<=3 & !missing(cf87ca714_w1) mi passive: replace pce5=1 if cf87ca714_w1>=4 & !missing(cf87ca714_w1)

mi passive: gen pce5a=0 if cf87ca714_w1<=4 & !missing(cf87ca714_w1) mi passive: replace pce5a=1 if cf87ca714_w1>=5 & !missing(cf87ca714_w1)

mi passive: gen pce5_lse_cat4=4 if lse_cat2e==1 & pce5==1	mi passive: replace pce5_lse_cat4=3 if lse_cat2e==0 & pce5==1	mi passive: replace pce5_lse_cat4=2 if lse_cat2e==1 & pce5==0	mi passive: replace pce5_lse_cat4=1 if lse_cat2e==0 & pce5==0 save ymh_mi_be_con.dta,replace

*****Model estimates** use ymh_mi_be_con.dta,clear

**MI Table 1: binary exposures local myoutlist be foreach myo in `myoutlist' {

tempname myhandle

file open `myhandle' using ymh_table_`myo'_mi_con.txt, write replace file write `myhandle' "MI Table 1 Associations between positive/adverse experiences and outcomes at wave 2" _n file write `myhandle' _tab ("Model1 B (95%)") _tab ("Model2 B (95%)") _tab ("p1") _tab ("p2") _n

local myvlist cf87ca711_w1 m_fco m_ffu cf87ca714_w1 cf87ca712_w1 m_nc chyrbsbb_w1 ph_w1 local myclist1 i.chsex_w1 chage_w1 i.pedulevel i.pemply_a i.pmar_b i.htype2_2 i.hhincome_2nsgp

local myclist2 i.chsex_w1 chage_w1 chqcf87`myo'_w1 i.pedulevel i.pemply_a i.pmar_b i.htype2_2 i.hhincome_2nsgp

file write `myhandle' "chqcf87`myo'" _n	foreach myv in `myvlist' {	file write `myhandle' "`myv'"

mi estimate, irr cmdok: poisson chqcf87`myo'_w2 `myv' [pweight = weights], vce(cl rhid)

matrix define myresults=r(table)	local myn: display %3.0f e(N)	local mymd: display %5.2f myresults[1,1]	local mypvalue1: display %5.3f myresults[4,1]	local mylci: display %5.2f myresults[5,1]	local myuci: display %5.2f myresults[6,1]	local mci="`mymd'" + " (" + "`mylci'"+ ", " + "`myuci'" + ")" + " [N=" + "`myn'" + "]"	file write `myhandle' _tab ("`mci'")

mi estimate, irr cmdok: poisson chqcf87`myo'_w2 `myv' `myclist1' [pweight = weights], vce(cl rhid)

matrix define myresults=r(table)

local myn: display %3.0f e(N)	local mymd: display %5.2f myresults[1,1]	local mypvalue2: display %5.3f myresults[4,1]	local mylci: display %5.2f myresults[5,1]	local myuci: display %5.2f myresults[6,1]	local mci="`mymd'" + " (" + "`mylci'"+ ", " + "`myuci'" + ")" + " [N=" + "`myn'" + "]"	file write `myhandle' _tab ("`mci'")

mi estimate, irr cmdok: poisson chqcf87`myo'_w2 `myv' `myclist2' [pweight = weights], vce(cl rhid)

matrix define myresults=r(table)	local myn: display %3.0f e(N)	local mymd: display %5.2f myresults[1,1]	local mypvalue3: display %5.3f myresults[4,1]	local mylci: display %5.2f myresults[5,1]	local myuci: display %5.2f myresults[6,1]	local mci="`mymd'" + " (" + "`mylci'"+ ", " + "`myuci'" + ")" + " [N=" + "`myn'" + "]"	file write `myhandle' _tab ("`mci'")	file write `myhandle' _tab ("`mypvalue1'") _tab ("`mypvalue2'") _tab ("`mypvalue3'")	file write `myhandle' _n	}/*close of the myvar loop*/

file close `myhandle' di as txt `"(Results are in {browse "ymh_table_`myo'_mi_con.txt"})"' }/*close of the myout loop*/

*MI Table2 lse_cat4 local myoutlist be foreach myo in `myoutlist' { tempname myhandle file open `myhandle' using ymh_table2_`myo'_lse_cat4_mi_con.txt, write replace file write `myhandle' "Table 2 Associations between stressful experiences and outcomes at wave 2" _n

local myvlist i.lse_cat4 local myclist1 i.chsex_w1 chage_w1 i.pedulevel i.pemply_a i.pmar_b i.htype2_2 i.hhincome_2nsgp local myclist2 i.chsex_w1 chage_w1 chqcf87`myo'_w1 i.pedulevel i.pemply_a i.pmar_b i.htype2_2 i.hhincome_2nsgp

file write `myhandle' "chqcf87`myo'" _n	foreach myv in `myvlist' {	file write `myhandle' "`myv'"

mi estimate, irr cmdok: poisson chqcf87`myo'_w2 `myv' [pweight = weights], vce(cl rhid)

matrix define myresults=r(table)	local myn: display %3.0f e(N)	local mymd: display %5.2f myresults[1,2]	local mypvalue: display %5.3f myresults[4,2]	local mylci: display %5.2f myresults[5,2]	local myuci: display %5.2f myresults[6,2]	local mci="`mymd'" + " (" + "`mylci'"+ ", " + "`myuci'" + ")" + " [N=" + "`myn'" + "]"	file write `myhandle' _tab ("`mci'") _tab ("`mypvalue'") _n

local myn: display %3.0f e(N)

local mymd: display %5.2f myresults[1,3]	local mypvalue: display %5.3f myresults[4,3]	local mylci: display %5.2f myresults[5,3]	local myuci: display %5.2f myresults[6,3]	local mci="`mymd'" + " (" + "`mylci'"+ ", " + "`myuci'" + ")" + " [N=" + "`myn'" + "]"	file write `myhandle' _tab ("`mci'") _tab ("`mypvalue'") _n

local myn: display %3.0f e(N)

local mymd: display %5.2f myresults[1,4]	local mypvalue: display %5.3f myresults[4,4]	local mylci: display %5.2f myresults[5,4]	local myuci: display %5.2f myresults[6,4]	local mci="`mymd'" + " (" + "`mylci'"+ ", " + "`myuci'" + ")" + " [N=" + "`myn'" + "]"	file write `myhandle' _tab ("`mci'") _tab ("`mypvalue'") _n

mi estimate, irr cmdok: poisson chqcf87`myo'_w2 `myv' `myclist1' [pweight = weights], vce(cl rhid)

matrix define myresults=r(table)

local myn: display %3.0f e(N)

local mymd: display %5.2f myresults[1,2]	local mypvalue: display %5.3f myresults[4,2]	local mylci: display %5.2f myresults[5,2]	local myuci: display %5.2f myresults[6,2]	local mci="`mymd'" + " (" + "`mylci'"+ ", " + "`myuci'" + ")" + " [N=" + "`myn'" + "]"	file write `myhandle' _tab ("`mci'") _tab ("`mypvalue'") _n

local myn: display %3.0f e(N)

local mymd: display %5.2f myresults[1,3]	local mypvalue: display %5.3f myresults[4,3]	local mylci: display %5.2f myresults[5,3]	local myuci: display %5.2f myresults[6,3]	local mci="`mymd'" + " (" + "`mylci'"+ ", " + "`myuci'" + ")" + " [N=" + "`myn'" + "]"	file write `myhandle' _tab ("`mci'") _tab ("`mypvalue'") _n

local myn: display %3.0f e(N)

local mymd: display %5.2f myresults[1,4]	local mypvalue: display %5.3f myresults[4,4]	local mylci: display %5.2f myresults[5,4]	local myuci: display %5.2f myresults[6,4]	local mci="`mymd'" + " (" + "`mylci'"+ ", " + "`myuci'" + ")" + " [N=" + "`myn'" + "]"	file write `myhandle' _tab ("`mci'") _tab ("`mypvalue'") _n

mi estimate, irr cmdok: poisson chqcf87`myo'_w2 `myv' `myclist2' [pweight = weights], vce(cl rhid)

matrix define myresults=r(table)

local myn: display %3.0f e(N)

local mymd: display %5.2f myresults[1,2]	local mypvalue: display %5.3f myresults[4,2]	local mylci: display %5.2f myresults[5,2]	local myuci: display %5.2f myresults[6,2]	local mci="`mymd'" + " (" + "`mylci'"+ ", " + "`myuci'" + ")" + " [N=" + "`myn'" + "]" file write `myhandle' _tab ("`mci'") _tab ("`mypvalue'") _n

local myn: display %3.0f e(N)

local mymd: display %5.2f myresults[1,3]	local mypvalue: display %5.3f myresults[4,3]	local mylci: display %5.2f myresults[5,3]	local myuci: display %5.2f myresults[6,3]	local mci="`mymd'" + " (" + "`mylci'"+ ", " + "`myuci'" + ")" + " [N=" + "`myn'" + "]"	file write `myhandle' _tab ("`mci'") _tab ("`mypvalue'") _n

local myn: display %3.0f e(N)

local mymd: display %5.2f myresults[1,4]	local mypvalue: display %5.3f myresults[4,4]	local mylci: display %5.2f myresults[5,4]	local myuci: display %5.2f myresults[6,4]	local mci="`mymd'" + " (" + "`mylci'"+ ", " + "`myuci'" + ")" + " [N=" + "`myn'" + "]"	file write `myhandle' _tab ("`mci'") _tab ("`mypvalue'")	file write `myhandle' _n	}/*close of the myvar loop*/

file close `myhandle' di as txt `"(Results are in {browse "ymh_table2_`myo'_lse_cat4_mi_con.txt"})"'	}/*close of the myout loop*/

**MI Table interaction use ymh_mi_be_con.dta,clear tempname myhandle file open `myhandle' using ymh_table11a_mi_be_con.txt, write replace

file write `myhandle' "Table 11a Interactions between positive and stressful experiences using poisson regression" _n

file write `myhandle' _tab ("Model1 B (95%)") _tab ("Model2 B (95%)") _tab ("Model3 B (95%)") _tab ("p1") _tab ("p2") _tab ("p3") _n

local myoutlist chqcf87be_w2 local myvlist2 cf87ca711_w1 m_fco m_ffu cf87ca714_w1 cf87ca712_w1 m_nc local myvlist lse_cat2e chyrbsbb_w1 ph_w1

local myclist1 i.chsex_w1 chage_w1 i.pedulevel i.pemply_a i.pmar_b i.htype2_2 i.hhincome_2nsgp local myclist2 i.chsex_w1 chage_w1 chqcf87be_w1 i.pedulevel i.pemply_a i.pmar_b i.htype2_2 i.hhincome_2nsgp

foreach myo in `myoutlist' {	file write `myhandle' "`myo'" _n

foreach myv in `myvlist' {	foreach myv2 in `myvlist2' {

*file write `myhandle' "`myv'" _n

mi estimate, irr cmdok: poisson `myo' i.`myv'##c.`myv2' [pweight = weights],vce(cl rhid)

matrix define myresults=r(table)	local myn1: display %3.0f e(N)	local mymd1: display %5.2f myresults[1,2]	local mypvalue1: display %5.3f myresults[4,2]	local mylci1: display %5.2f myresults[5,2]	local myuci1: display %5.2f myresults[6,2]	local mci1="`mymd1'" + " (" + "`mylci1'"+ ", " + "`myuci1'" + ")"

local myn2: display %3.0f e(N)	local mymd2: display %5.2f myresults[1,3]	local mypvalue2: display %5.3f myresults[4,3]	local mylci2: display %5.2f myresults[5,3]	local myuci2: display %5.2f myresults[6,3]	local mci2="`mymd2'" + " (" + "`mylci2'"+ ", " + "`myuci2'" + ")"

local myn3: display %3.0f e(N)	local mymd3: display %5.2f myresults[1,5]	local mypvalue3: display %5.3f myresults[4,5]	local mylci3: display %5.2f myresults[5,5]	local myuci3: display %5.2f myresults[6,5]	local mci3="`mymd3'" + " (" + "`mylci3'"+ ", " + "`myuci3'" + ")"

mi estimate, irr cmdok: poisson `myo' i.`myv'##c.`myv2' `myclist1' [pweight = weights], vce(cl rhid)

matrix define myresults=r(table)	local myn4: display %3.0f e(N)	local mymd4: display %5.2f myresults[1,2]	local mypvalue4: display %5.3f myresults[4,2]	local mylci4: display %5.2f myresults[5,2]	local myuci4: display %5.2f myresults[6,2]	local mci4="`mymd4'" + " (" + "`mylci4'"+ ", " + "`myuci4'" + ")"

local myn5: display %3.0f e(N)	local mymd5: display %5.2f myresults[1,3]	local mypvalue5: display %5.3f myresults[4,3]	local mylci5: display %5.2f myresults[5,3]	local myuci5: display %5.2f myresults[6,3]	local mci5="`mymd5'" + " (" + "`mylci5'"+ ", " + "`myuci5'" + ")"

local myn6: display %3.0f e(N)	local mymd6: display %5.2f myresults[1,5]	local mypvalue6: display %5.3f myresults[4,5]	local mylci6: display %5.2f myresults[5,5]	local myuci6: display %5.2f myresults[6,5]	local mci6="`mymd6'" + " (" + "`mylci6'"+ ", " + "`myuci6'" + ")"

mi estimate, irr cmdok: poisson `myo' i.`myv'##c.`myv2' `myclist2' [pweight = weights], vce(cl rhid)

matrix define myresults=r(table)	local myn7: display %3.0f e(N)	local mymd7: display %5.2f myresults[1,2]	local mypvalue7: display %5.3f myresults[4,2]	local mylci7: display %5.2f myresults[5,2]	local myuci7: display %5.2f myresults[6,2]	local mci7="`mymd7'" + " (" + "`mylci7'"+ ", " + "`myuci7'" + ")"

local myn8: display %3.0f e(N)	local mymd8: display %5.2f myresults[1,3] local mypvalue8: display %5.3f myresults[4,3]	local mylci8: display %5.2f myresults[5,3]	local myuci8: display %5.2f myresults[6,3]	local mci8="`mymd8'" + " (" + "`mylci8'"+ ", " + "`myuci8'" + ")"

local myn9: display %3.0f e(N)	local mymd9: display %5.2f myresults[1,5]	local mypvalue9: display %5.3f myresults[4,5]	local mylci9: display %5.2f myresults[5,5]	local myuci9: display %5.2f myresults[6,5]	local mci9="`mymd9'" + " (" + "`mylci9'"+ ", " + "`myuci9'" + ")"

*file write `myhandle' "`myv'" _tab ("`mci1'") _tab ("`mci4'") _tab ("`mci7'") _tab ("`mypvalue1'") _tab ("`mypvalue4'") _tab ("`mypvalue7'") _n

*file write `myhandle' "`myv2'" _tab ("`mci2'") _tab ("`mci5'") _tab ("`mci8'") _tab ("`mypvalue2'")

_tab ("`mypvalue5'") _tab ("`mypvalue8'") _n

file write `myhandle' "`myv'*`myv2'" _tab ("`mci3'") _tab ("`mci6'") _tab ("`mci9'") _tab ("`mypvalue3'") _tab ("`mypvalue6'") _tab ("`mypvalue9'") _n

*file write `myhandle' _n

}/*close of the myvar loop*/

}/*close of the myvar loop*/

}/*close of the myout loop*/ file close `myhandle' di as txt `"(Results are in {browse "ymh_table11a_mi_be_con.txt"})"'

*****Margins for figure 3 local myoutlist chqcf87be_w2 local myvlist pce5 local myvlist2 lse_cat2e local myclist2 i.chsex_w1 chage_w1 chqcf87be_w1 i.pedulevel i.pemply_a i.pmar_b i.htype2_2 i.hhincome_2nsgp

foreach myo in `myoutlist' {

foreach myv in `myvlist' {

foreach myv2 in `myvlist2' {

putexcel set ymh_mi_margin_`myo'_`myv'_`myv2'_myclist1, replace sheet("Table 1")

foreach i of numlist 1 {

use ymh_mi_be_con.dta,clear	mi extract `i'

poisson `myo' i.`myv'##i.`myv2' `myclist2' [pweight = weights], vce(cl rhid)

margins `myv'#`myv2'	matrix irr1=r(table)[1,1]'	matrix irr_se1=r(table)[2,1]'	matrix irr_lci1=r(table)[5,1]'	matrix irr_uci1=r(table)[6,1]'	putexcel a`i'=matrix(irr1), colnames	putexcel b`i'=matrix(irr_se1), colnames	putexcel c`i'=matrix(irr_lci1), colnames	putexcel d`i'=matrix(irr_uci1), colnames

matrix irr2=r(table)[1,2]'	matrix irr_se2=r(table)[2,2]'	matrix irr_lci2=r(table)[5,2]'	matrix irr_uci2=r(table)[6,2]'	putexcel e`i'=matrix(irr2), colnames	putexcel f`i'=matrix(irr_se2), colnames	putexcel g`i'=matrix(irr_lci2), colnames	putexcel h`i'=matrix(irr_uci2), colnames

matrix irr3=r(table)[1,3]'	matrix irr_se3=r(table)[2,3]'	matrix irr_lci3=r(table)[5,3]'	matrix irr_uci3=r(table)[6,3]'	putexcel i`i'=matrix(irr3), colnames	putexcel j`i'=matrix(irr_se3), colnames	putexcel k`i'=matrix(irr_lci3), colnames	putexcel l`i'=matrix(irr_uci3), colnames

matrix irr4=r(table)[1,4]'

matrix irr_se4=r(table)[2,4]'	matrix irr_lci4=r(table)[5,4]'	matrix irr_uci4=r(table)[6,4]'	putexcel m`i'=matrix(irr4), colnames	putexcel n`i'=matrix(irr_se4), colnames	putexcel o`i'=matrix(irr_lci4), colnames	putexcel p`i'=matrix(irr_uci4), colnames

}

forvalues i=3/21 {	use ymh_mi_be_con.dta,clear	local j=`i'-1	mi extract `j'

poisson `myo' i.`myv'##i.`myv2' `myclist2' [pweight = weights], vce(cl rhid)

margins `myv'#`myv2'	matrix irr1=r(table)[1,1]'	matrix irr_se1=r(table)[2,1]'	matrix irr_lci1=r(table)[5,1]'	matrix irr_uci1=r(table)[6,1]'	putexcel a`i'=matrix(irr1)	putexcel b`i'=matrix(irr_se1)	putexcel c`i'=matrix(irr_lci1)	putexcel d`i'=matrix(irr_uci1)

matrix irr2=r(table)[1,2]'	matrix irr_se2=r(table)[2,2]'	matrix irr_lci2=r(table)[5,2]'	matrix irr_uci2=r(table)[6,2]'	putexcel e`i'=matrix(irr2)

putexcel f`i'=matrix(irr_se2)	putexcel g`i'=matrix(irr_lci2)	putexcel h`i'=matrix(irr_uci2) matrix irr3=r(table)[1,3]'	matrix irr_se3=r(table)[2,3]'	matrix irr_lci3=r(table)[5,3]'	matrix irr_uci3=r(table)[6,3]'	putexcel i`i'=matrix(irr3)	putexcel j`i'=matrix(irr_se3)	putexcel k`i'=matrix(irr_lci3)	putexcel l`i'=matrix(irr_uci3)

matrix irr4=r(table)[1,4]'	matrix irr_se4=r(table)[2,4]'	matrix irr_lci4=r(table)[5,4]'	matrix irr_uci4=r(table)[6,4]'	putexcel m`i'=matrix(irr4)	putexcel n`i'=matrix(irr_se4)	putexcel o`i'=matrix(irr_lci4)	putexcel p`i'=matrix(irr_uci4)

}

putexcel a1=("irr1")	putexcel b1=("irr_se1")	putexcel c1=("irr_lci1")	putexcel d1=("irr_uci1")

putexcel e1=("irr2")	putexcel f1=("irr_se2")	putexcel g1=("irr_lci2")	putexcel h1=("irr_uci2")

putexcel i1=("irr3")

putexcel j1=("irr_se3") putexcel k1=("irr_lci3") putexcel l1=("irr_uci3") putexcel m1=("irr4") putexcel n1=("irr_se4") putexcel o1=("irr_lci4") putexcel p1=("irr_uci4")

di as txt `"(output written to {browse "ymh_mi_margin_`myo'_`myv'_`myv2'_myclist1.xlsx"})"'

}

}

}

*****Pool margins for figure 3 local myvlist pce5 local myvlist2 lse_cat2e local myoutlist chqcf87be_w2

foreach myo in `myoutlist' { foreach myv in `myvlist'{ foreach myv2 in `myvlist2'{	import excel "ymh_mi_margin_`myo'_`myv'_`myv2'_myclist1.xlsx", first clear gen num=_n

reshape long irr irr_se irr_lci irr_uci, i(num) j(group)

****pooled irr bys group: egen mean_irr=mean(irr)

**pooled se

**https://bookdown.org/mwheymans/bookmi/rubins-rules.html bys group: egen var_wt=mean(irr_se*irr_se) bys group: gen var_bt=(irr-mean_irr)*(irr-mean_irr) bys group: egen var_bt1=sum(var_bt) bys group: replace var_bt=var_bt1/19 drop var_bt1

bys group: gen var_total=(var_wt+var_bt)+var_bt/20

bys group: gen se_pooled=sqrt(var_total)

*pooled confidence interval bys group: gen lci_pooled=mean_irr+invnormal(0.025)*se_pooled bys group: gen uci_pooled=mean_irr-invnormal(0.025)*se_pooled

keep if num==1 keep mean_irr lci_pooled uci_pooled save ymh_mi_margin_`myo'_`myv'_`myv2'_myclist1_pooled.dta, replace

export excel mean_irr lci_pooled uci_pooled using "ymh_mi_margin_`myo'_`myv'_`myv2'_myclist1_pooled.xlsx", first(var) keepcellfmt replace

}

}

}

*Visualisation (Figure 3) import excel using ymh_mi_margin_chqcf87be_w2_pce5_lse_cat2e_myclist1_pooled_edit.xlsx, first clear

twoway (connect mean_irr pce if lse==0 , ms(O) pstyle(p1) lpattern(solid)) || ///

(rcap lci_pooled uci_pooled pce if lse==0 , pstyle(p1) lpattern(dash)) || ///

(connect mean_irr pce if lse==1, ms(Oh) pstyle(p2) lpattern(solid)) || ///	(rcap lci_pooled uci_pooled pce if lse==1 , pstyle(p2) lpattern(dash)) ||, ///	title("") ytitle("Behavioural well-being") xtitle("Positive peer relationship", size(small)) ///	xscale(range(-0.3 1.4)) xlabel(0 "No" 1 "Yes",labs(small)) yscale (range(84 94)) ///

legend(pos(5) ring(0) col(2) label(1 "Stressful life events <2") label(3 "Stressful life events {&ge}3")) legend(order(1 3) size(small)) scheme(s1color)

graph save ymh_lse_pce5_a.gph,replace

**References**

## Shi B, Choirat C, Coull BA, VanderWeele TJ and Valeri L (2021) CMAverse: A Suite of Functions for

Reproducible Causal Mediation Analyses. *Epidemiology* **32**(5)**,** e20-e22.

**VanderWeele TJ and Vansteelandt S** (2014) Mediation Analysis with Multiple Mediators. *Epidemiol*

*Methods* **2**(1)**,** 95-115.
